# Vertically stratified methane, nitrogen and sulphur cycling and coupling mechanisms in mangrove sediment microbiomes

**DOI:** 10.1186/s40168-023-01501-5

**Published:** 2023-04-05

**Authors:** Lu Qian, Xiaoli Yu, Hang Gu, Fei Liu, Yijun Fan, Cheng Wang, Qiang He, Yun Tian, Yisheng Peng, Longfei Shu, Shanquan Wang, Zhijian Huang, Qingyun Yan, Jianguo He, Guangli Liu, Qichao Tu, Zhili He

**Affiliations:** 1 Environmental Microbiomics Research Center, School of Environmental Science and Engineering, Southern Marine Science and Engineering Guangdong Laboratory (Zhuhai), State Key Laboratory of Biocontrol, Sun Yat-Sen University, Guangzhou, 510006 China; 2 Department of Civil and Environmental Engineering, the University of Tennessee, Knoxville, TN 37996 USA; 3 Key Laboratory of the Ministry of Education for Coastal and Wetland Ecosystems, School of Life Sciences, Xiamen University, Xiamen, 361005 China; 4 School of Marine Science, Sun Yat-Sen University, Zhuhai, 519080 China; 5 School of Life Science, Sun Yat-Sen University, Guangzhou, 510275 China; 6 Institute of Marine Science and Technology, Shandong University, Qingdao, 266237 China

**Keywords:** Vertical distribution, Metagenome sequencing analysis, Mangrove sediment, Methane/nitrogen/sulphur cycling, Metagenome-assembled genome, Coupling mechanism

## Abstract

**Background:**

Mangrove ecosystems are considered as hot spots of biogeochemical cycling, yet the diversity, function and coupling mechanism of microbially driven biogeochemical cycling along the sediment depth of mangrove wetlands remain elusive. Here we investigated the vertical profile of methane (CH_4_), nitrogen (N) and sulphur (S) cycling genes/pathways and their potential coupling mechanisms using metagenome sequencing approaches.

**Results:**

Our results showed that the metabolic pathways involved in CH_4_, N and S cycling were mainly shaped by pH and acid volatile sulphide (AVS) along a sediment depth, and AVS was a critical electron donor impacting mangrove sediment S oxidation and denitrification. Gene families involved in S oxidation and denitrification significantly (*P* < 0.05) decreased along the sediment depth and could be coupled by S-driven denitrifiers, such as *Burkholderiaceae* and *Sulfurifustis* in the surface sediment (0–15 cm). Interestingly, all S-driven denitrifier metagenome-assembled genomes (MAGs) appeared to be incomplete denitrifiers with nitrate/nitrite/nitric oxide reductases (Nar/Nir/Nor) but without nitrous oxide reductase (Nos), suggesting such sulphide-utilizing groups might be an important contributor to N_2_O production in the surface mangrove sediment. Gene families involved in methanogenesis and S reduction significantly (*P* < 0.05) increased along the sediment depth. Based on both network and MAG analyses, sulphate-reducing bacteria (SRB) might develop syntrophic relationships with anaerobic CH_4_ oxidizers (ANMEs) by direct electron transfer or zero-valent sulphur, which would pull forward the co-existence of methanogens and SRB in the middle and deep layer sediments.

**Conclusions:**

In addition to offering a perspective on the vertical distribution of microbially driven CH_4_, N and S cycling genes/pathways, this study emphasizes the important role of S-driven denitrifiers on N_2_O emissions and various possible coupling mechanisms of ANMEs and SRB along the mangrove sediment depth. The exploration of potential coupling mechanisms provides novel insights into future synthetic microbial community construction and analysis. This study also has important implications for predicting ecosystem functions within the context of environmental and global change.

Video Abstract

**Supplementary Information:**

The online version contains supplementary material available at 10.1186/s40168-023-01501-5.

## Background

Mangroves, one of the typical blue carbon ecosystems with the methane (CH_4_) emission around 279.17 μmol m^−2^ day^−1^ on Earth [1], have tremendous ecological importance in the global carbon (C), nitrogen (N) and sulphur (S) cycles. The metabolic function of biogeochemical cycling processes is determined by biotic (e.g. microorganisms, microbial genes/pathways) and abiotic (e.g. pH, electron donors, electron acceptors) factors as well as their interactions [2–4]. Microbes are the engine that drives these biogeochemical processes and have profound effects on ecosystem functions [5], such as CH_4_ emissions [6, 7], N transformation and removal [8, 9], and S reduction and oxidation [10, 11]. The ‘hot spot’ of microbially driven biogeochemical cycling could create an eco-buffer zone for shoreline protection, nutrient filtering, C storage and climate regulation [12].

Mangrove sediments form a dynamic environment and create an ideal natural gradient along the depth due to periodical tide movements, and fine gradients of physicochemical conditions, thus distinct microbial functions are observed at such land-sea interfaces [13–15]. For example, it was reported that denitrifiers had high activities and were responsible for major (~80%) N loss in the surface coastal sediment [16]. The high denitrification rate observed in the presence of O_2_ could be explained by the adaptation of denitrifying bacteria to tidally induced recurrent redox oscillations in permeable sediments [17]. In the mangrove sediment, sulphide, H_2_ and organic C compounds are common electron donors [18, 19] and oxygen, sulphate, nitrate and Fe(III) are major electron acceptors, which co-exist along the sediment depth [20]. The vertical distribution of electron donors and acceptors showed an obvious impact on microbial functions, such as anaerobic oxidation of methane (AOM) and sulphate reduction [21, 22]. Recently, we found that N_2_ fixation rate increased while the diversity of diazotrophic communities decreased along the depth of mangrove sediments largely due to the vertical variation of salinity [23]. However, most of such studies were focused exclusively on the top 20 cm of mangrove sediments, which was considered as the layer with the greatest microbial diversity, biomass and activity [6, 20, 24]. Therefore, a comprehensive profile of CH_4_, N and S cycling genes/pathways along a vertical gradient of mangrove sediments is crucial for predicting mangrove ecosystem functions.

Generally, microbes in natural ecosystems do not exist independently, and they interact with each other to form complex microbial communities and couple with different biogeochemical cycling processes. Microbial interactions have been well investigated in the laboratory [25] or in several natural ecosystems such as lakes [26, 27] and marine sediments [28, 29]. For example, nitrate reduction could be coupled with S oxidation by a single organism [30] or syntrophic microbial consortia [28]; anaerobic CH_4_ oxidizers (ANMEs) and sulphate-reducing bacteria (SRB) or S-disproportionating bacteria were found to be syntrophic partners responsible for anaerobic CH_4_ oxidation and sulphate reduction in anoxic CH_4_-rich sediments [29, 31]. However, such microbial interactions in mangrove sediments remain poorly understood due to their extremely high diversity and the as-yet uncultivated status of major environmental microorganisms. Metagenomic analysis provides the possibility to explore the potential microbial interactions without the limitation in terms of uncultivated microbes [26, 32]. Thus, it could be important to understand the microbially driven CH_4_, N and S cycling as well as their coupling mechanisms by metagenome sequencing analysis, which may enable us to develop novel strategies for enhancing mangrove ecosystem functions and mitigating climate change.

In this study, we aimed to understand the vertical distribution of microbially driven CH_4_, N and S cycling and their potential coupling mechanisms in mangrove ecosystems. Due to the vertical changes of nutrients (e.g. total C, total N), electron status (e.g. acid volatile sulphide, SO_4_^2−^), and/or environmental conditions (e.g. pH, temperature) [6, 33], we hypothesized that the overall functional diversity of sedimentary microbial communities would decrease, their functional composition and interactions would shift as the depth increased, and S cycling microbes could play an important role in coupling biogeochemical cycling processes due to their metabolic versatility in response to mangrove sedimentary nutrients, electron acceptors and donors, and environmental conditions at different depths [34, 35]. To test the hypotheses, we collected 0–100-cm sediment samples from the Qi’ao Mangrove Reserve in Zhuhai, China. The vertical profile of CH_4_, N and S cycling genes/pathways was analysed by metagenome sequencing approaches, and their possible coupling mechanisms among CH_4_, N and S cycling were explored. This study provides a comprehensive perspective of vertical distribution of CH_4_, N and S metabolisms at a fine spatial scale and improves our understanding of their possible coupling mechanisms in mangrove sediments.

## Methods

### Sample collection and processing

The sampling site is located at the Qi’ao Mangrove Reserve (22.42° N, 113.63° E) in Zhuhai, China. Five 1-m plunger cores were collected as replicates from a mangrove habitat dominated by *Kandelia obovata* in December 2019 (Additional file 1: Fig. S1). The sediment cores were divided into 10 depths (i.e. 0–5, 5–10, 10–15, 15–20, 20–30, 30–40, 40–50, 50–60, 60–80 and 80–100 cm), resulting in a total of 50 samples. These samples were stored in a portable cooler at 4°C and transported to the laboratory within 24 h. Each sample was divided into two subsamples: one was immediately stored at −80°C for DNA extraction, and the other was stored at 4°C for physicochemical analysis.

### Sediment physicochemical properties analysis

Temperature and pH of sediment were measured in situ by a hand-held metre (Extech Instruments, A FLIR Company, USA). Salinity was determined with 2.0 g dry sediment in a 1:5 sediment/water suspension with a salinity metre (EUTECH SALT6þ, Thermo Scientific, USA). Soil ammonium (NH_4_^+^), nitrite (NO_2_^−^) and nitrate (NO_3_^−^) were extracted using 2 M KCl and measured with a multimode microplate reader (Varioskan LUX, Thermo Scientific, USA). Porewater sulphate (SO_4_^2−^) concentration was measured with porewater extracted from 10.0 g fresh sediment by an ion chromatography (Dionex ICS-1100, Thermo Scientific, USA). Acid volatile sulphide (AVS) was treated with acid to release H_2_S and measured by iodometric titration method [36]. S^0^ was measured with a high-performance liquid chromatograph (Agilent 1260 Infinity II, Agilent, Germany). Sediment samples for measuring the total carbon (TC), total nitrogen (TN) and total sulphur (TS) were dried at 65°C until reaching a constant weight, finely ground, and then measured by an elemental analyser (Vario TOC, Elemental, Germany).

### Sediment DNA extraction and shotgun metagenome sequencing

Sediment microbial community DNA was extracted with 5.0 g fresh sediment using a modified freeze-grinding plus sodium dodecyl sulphate (SDS) lysis method as described previously [37], and purified by Power Soil DNA Isolation Kit (Mo Bio Laboratories, Carlsbad, California, USA). DNA quality was assessed by a Nanodrop (NanoDrop One, Thermo Scientific, USA), all the DNA kept in subsequent experiment with the ratios of 260/280 nm and 260/230 nm were around 1.8 and above 1.7, respectively. The final DNA concentration was quantified by a fluorescent method (Qubit 4 Fluorometer, Thermo Scientific, USA).

All 50 samples were subjected to shotgun metagenome sequencing. Dual-indexed DNA sequencing libraries were constructed using NEBNext® UltraTM DNA Library Prep Kit for Illumina (NEB, USA) according to the manufacturer’s recommendations. Prepared library DNA concentrations were determined with a Qubit HS DNA assay and libraries were run on a High Sensitivity DNA chip using the Agilent 2100 Bioanalyzer to determine library average insert sizes. After cluster generation was performed on a cBot Cluster Generation System, paired-end reads (PE150) were sequenced in Guangdong Magigene Biotechnology Co., Ltd. with an Illumina HiSeq2500 platform. For each sample, approximately 10 Gb of metagenome sequencing data (33,504,696 to 67,951,834 reads per sample) (Additional file 1: Table S1) was generated after adapter trimming and sequence filtering. Specifically, the paired reads with more than 10% unknown bases (‘N’ bases) or more than 50% of low-quality bases (quality value ≤ 5) in any read were removed [38].

### Read-based functional and taxonomical analysis

The raw data were further quality-trimmed with BBDuk (k=28 mink=12 trimq=20 minlength=70) (https://jgi.doe.gov/data-and-tools/bbtools/bb-tools-user-guide/bbduk-guide/), and the sequence quality was assessed using FastQC (http://www.bioinformatics.babraham.ac.uk/projects/fastqc/). Qualified metagenomic paired-end reads were merged using PEAR (options: -p 0.001) [39], and the merged sequences were used for functional annotation and taxonomic assignments.

Gene prediction was carried out by Prodigal (https://github.com/hyattpd/Prodigal), which could predict high-quality gene fragments from merged short reads. The predicted gene fragments were searched against KEGG (http://www.genome.jp/kegg/pathway), MCycDB [40], SCycDB [41] and NCycDB [42] reference databases using DIAMOND [43] (options: -k 1 -e 1E-5-sensitive) for functional annotation. All samples were rarefied to the same sequencing depth (6,779,669 sequences per samples) by random resampling. The relative abundance of genes was defined as the number of gene sequence reads. The obtained functional profiles of KO terms, CH_4,_ N or S cycling gene families were used for the subsequent analysis.

The overall taxonomic assignments of microbial communities were assessed using the phylogenetic annotation of merged reads with Kraken2 [44]. The genetic potential of a specific pathway was analysed following the key gene families involved in CH_4_, N and S cycling. The reads related to each key gene family were retrieved using SeqKit [45]. The taxonomic placement of each read was inferred for searching the Kraken2 database. The representatives of key gene families were further analysed, including *mcrA* for methanogenesis and *pmoA* for aerobic oxidation of CH_4_; *nrfA* for dissimilatory nitrate reduction (DNRA), *nifH* for N fixation, *narG*, *nirS*, *nirK*, *norB* and *nosZ* for denitrification, and *hzsA* for anammox; *soxB*, *sqr* and *fccB* for S oxidation, *asrB* for S reduction and *sat*, *aprA*, *dsrA* and *dsrB* for dissimilatory sulphate reduction or S oxidation.

### Metagenome sequence assembly, binning and genome annotations

Quality-filtered reads were assembled to contigs with MEGAHIT [46] at the default mode according to the MetaWRAP analysis workflow. Open reading frame (ORF) fragments were predicted using Prodigal and searched against MCycDB [40], SCycDB [41] and NCycDB [42]. All target sequences were retrieved and taxonomically annotated using Kraken2. Their abundances were estimated using Salmon [47] and normalized into Transcripts Per Million (TPM) counts. Binning was conducted using MetaBAT2 (v2.12.1) [48] and the resulting bins were consolidated with the Bin_refinement module. The bins were dereplicated with dRep [49] with a secondary cluster ANI 99% (strain level). CheckM [50] was used to assessed the quality score (completeness – 5*contamination) of genome bins and only genomes with a quality score of ≥60 were retained [51, 52]. This resulted in a total of 77 high-quality dereplicated genome bins, hereafter referred to as metagenome-assembled genomes (MAGs). The relative abundances of MAGs in different samples were assessed with CoverM (https://github.com/wwood/CoverM). The taxonomy of MAGs was assigned with GTDB-Tk v1.3.0 reference data version r95 [53], and their gene predictions were performed using Prodigal, and the predicted genes were further annotated using METABOLIC [54] and eggNOG-mapper [55].

### Molecular ecological network construction and analysis

To explore how microbes and key genes involved in CH_4_, N and S cycling processes interact in the mangrove sediment, we constructed molecular ecological co-occurrence networks based on the microbes involved in CH_4_, N and S cycling. We grouped all 10 depths into three layers: surface (0–15 cm), middle (15–30 cm) and deep (30–100 cm) layers based on multiple response permutation procedure (MRPP), and analysis of similarity (ANOSIM) among functional profiles of microbial communities from different depths (Additional file 1: Table S2, Table S3). Three co-occurrence networks were constructed based on taxonomic profiles, which were generated from the taxonomy of all contigs involved in CH_4_/N/S cycling processes. The networks were constructed using the Molecular Ecological Network Analyses (MENA) pipeline [56], which implements Random Matrix Theory (RMT) to identify thresholds for constructing highly confident microbial ecological networks. Only taxa representing in half or more samples were selected to calculate the Spearman correlation coefficient (*r*), and the minimum threshold of *r* = 0.8 was automatically determined by the RMT approach. Network topological parameters were calculated by the MENA pipeline. The within-module connectivity (*Z*_*i*_) and among-module connectivity (*P*_*i*_) of each node were calculated to define their roles in the networks: peripherals (*Z*_*i*_ < 2.5, *P*_*i*_ < 0.62), connectors (*Z*_*i*_ < 2.5, *P*_*i*_>0.62), module hubs (*Z*_*i*_>2.5, *P*_*i*_ < 0.62) and network hubs (*Z*_*i*_>2.5, *P*_*i*_>0.62). The latter three were regarded as keystone taxa because of their important roles in network topology. The constructed microbial networks were visualized by Cytoscape [57].

### Statistical analysis

Following analyses were performed to test whether gene families display associations with environmental variables. First, Mantel tests were used to determine the linkage between environmental variables and CH_4_/N/S cycling gene abundances. Second, the maximal information coefficient (MIC) was estimated to capture diverse relationships between gene families and environmental factors [58], and relationships with MIC ≥0.4 were considered statistically significant. The MIC value significance was assessed using precomputed *P* values. And we used the percentage of genes that were significantly (MIC score ≥0.4) associated to an environmental variable among the total of genes involved in a pathway to estimate the importance of an environmental variable to a specific pathway. The α-diversity (Shannon index) of sediment microbial communities was calculated based on resampled functional and taxonomical profiles using the Galaxy pipeline (http://192.168.3.11:8080/), and their β-diversity was estimated using principal coordinates analysis (PCoA) based on the Bray–Curtis distance. ANOSIM and MRPP were used to evaluate the significance of compositional differences between functional diversity and sediment depths. Linear regression was used to explore the relationship between the abundance of key genes and sediment depths. One-way analysis of variance (ANOVA) was performed with the IBM SPSS 22 (SPSS Inc., USA) to compare the mean of sediment parameters, functional diversity and abundances among different depths. Spearman analysis was performed to estimate the correlation between key genes. All the statistical analyses were visualized using R packages including Minerva [59], reshape2 [60], rstatix [61], vegan [62], ggplot2 [63] and pheatmap [64] and GraphPad Prism 8.0.

## Results

### Vertical variation of physicochemical properties in mangrove sediments

We measured sediment pH, temperature, salinity, SO_4_^2−^, AVS, S^0^, NH_4_^+^, NO_2_^−^, NO_3_^−^, TC, TN and TS to characterize the vertical distribution of environmental factors in mangrove sediments (Additional file 1: Table S4). The results showed that pH and temperature significantly (*P* < 0.05; multiple comparison with one-way ANOVA analysis) increased with the depth, while salinity, TC, TN, SO_4_^2−^ and AVS showed an opposite trend. The sediment had higher concentrations of NH_4_^+^ (1800–4700 μg/L) relative to NO_3_^−^ (88.3–294.9 μg/L) or NO_2_^−^ (58.9–103.9 μg/L), and the maximum NH_4_^+^ concentration appeared at the 5–10 cm sediment depth. Additionally, the concentrations of S^0^ and TS did not change through the vertical profile.

### Environmental drivers of CH_4_, N and S cycling microbiomes

**Fig. 1:**
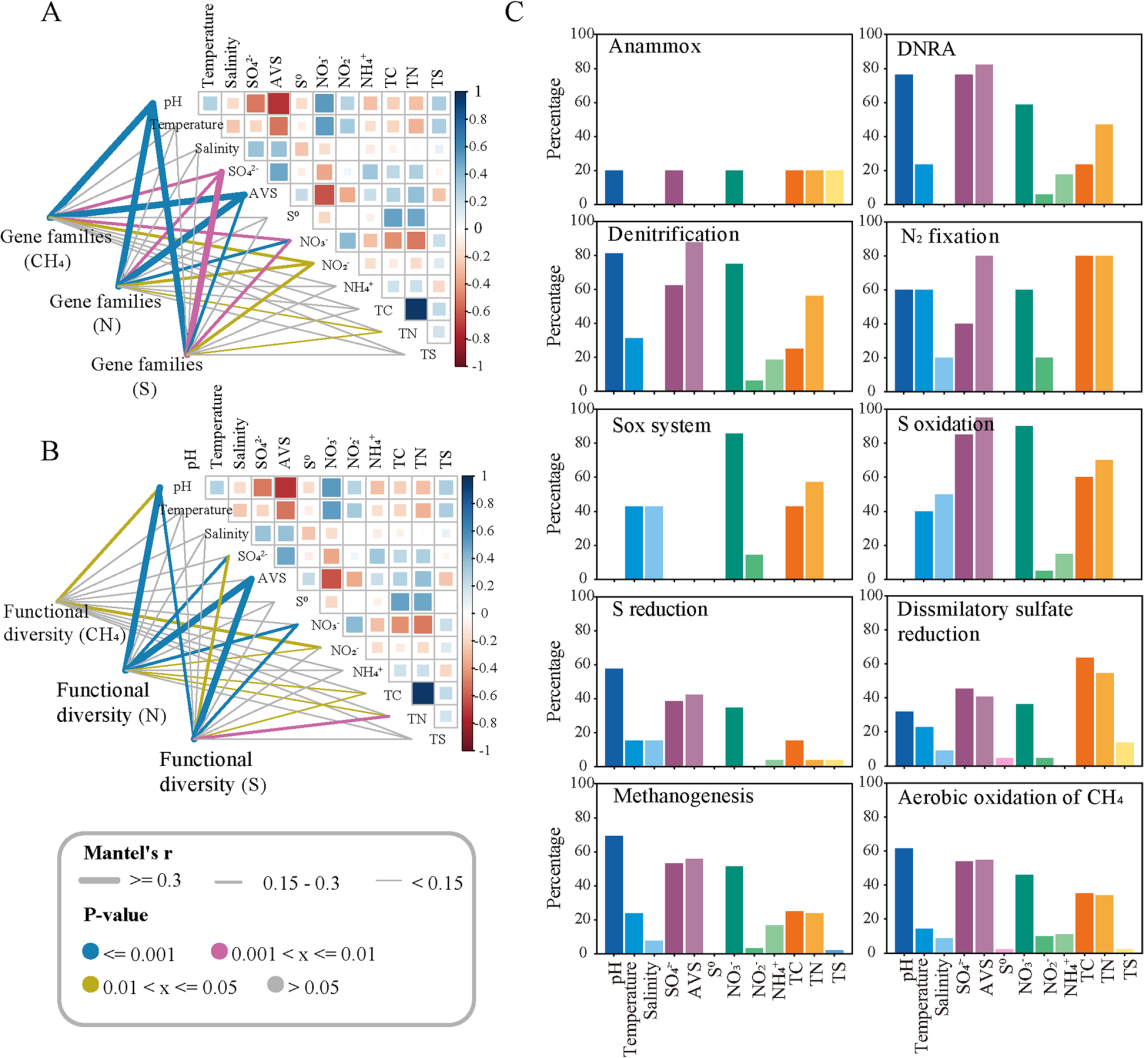
Associations between environmental factors and functions of mangrove sediment microbiomes. Mantel tests revealed the correlation between environmental factors and CH_4_/N/S cycling gene families (**A**), or functional diversity (**B**). **C** The importance of individual environmental factors to a specific metabolic pathway involved in CH_4_/N/S cycling was calculated using the maximal information coefficient (MIC) index. The percentage refers to the percentage of gene families involved in the specific metabolic pathway significantly driven by environmental factors. DNRA: dissimilatory nitrate reduction

To disentangle potential environmental drivers of microbiome functions in mangrove sediments, we performed Mantel tests and estimated the maximal information coefficient (MIC) value between physicochemical properties and the abundance of gene families involved in CH_4_/N/S cycling. The results showed that pH and AVS were the dominant factors shaping the functional diversity and composition of CH_4_, N and S cycling microbiomes, and followed by SO_4_^2−^ and NO_3_^−^ along the sediment depth (Fig. 1A, B). Specifically, pH showed significantly (*P* < 0.001) positive correlations with the relative abundance of functional genes involved in CH_4_, N and S cycling (Additional file 1: Table S5), and had a high importance in structuring these metabolic pathways in mangrove sediments, explaining 81.3% of the turnover of denitrification gene families, 76.5% for dissimilatory nitrate reduction (DNRA), 57.7% for S reduction, 69.2% for methanogenesis and 61.5% for aerobic oxidation of CH_4_ (Fig. 1C). Additionally, AVS was found to have the largest influences on denitrification and S oxidation, explaining 87.5 and 95.0%, respectively, while nitrate showed the greatest effect on Sox systems and S oxidation, explaining 85.7 and 90.0%, respectively. Collectively, the results revealed the importance of pH and AVS for driving the depth-dependent variability of microbially mediated CH_4_, N and S cycling processes, where AVS could be an important electron donor for nitrate reduction in mangrove sediments.

### An overview of metagenomic characteristics of mangrove sediment microbiomes along the sediment depth

**Fig. 2:**
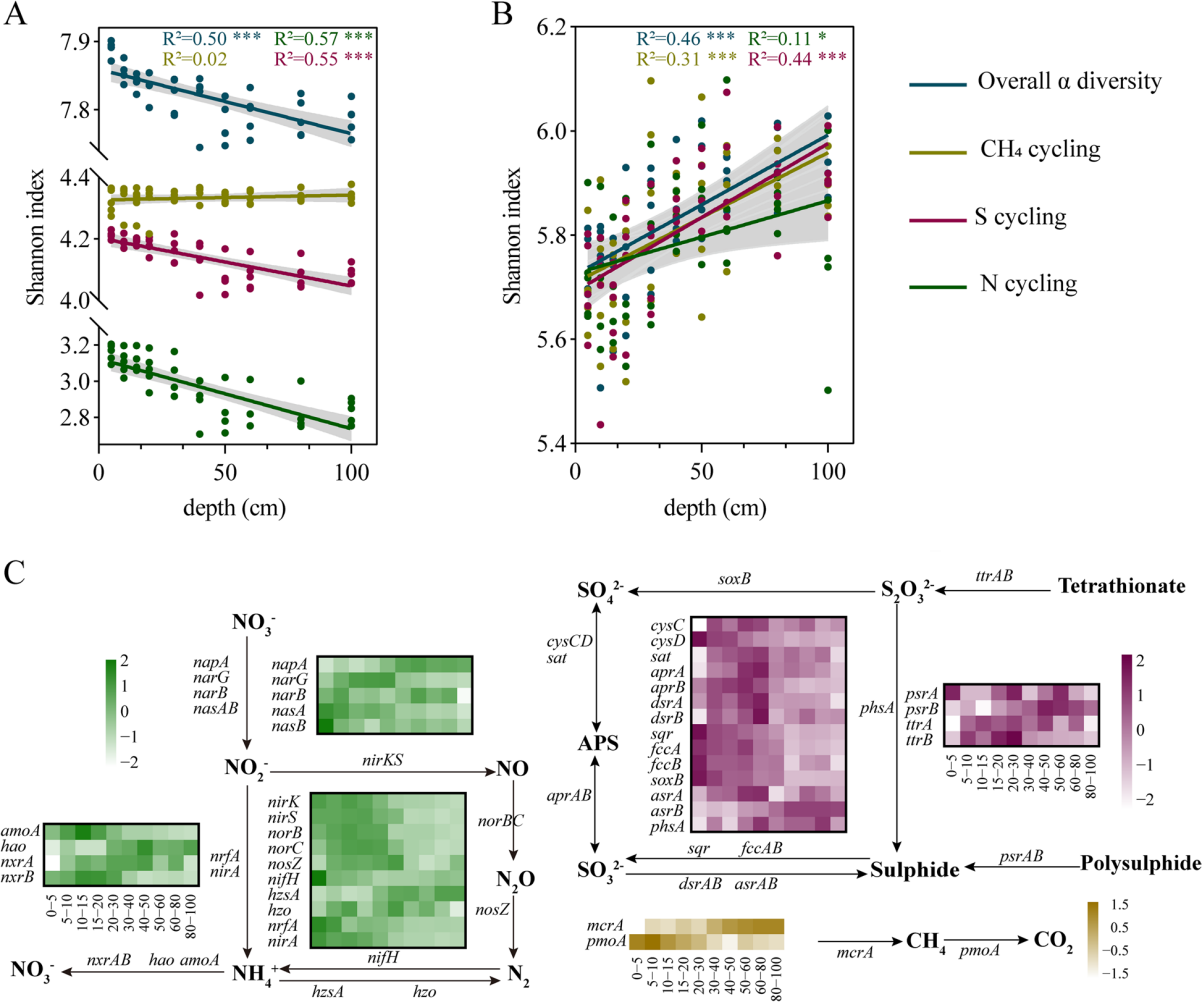
Depth variations of functional potentials of mangrove sediment microbiomes. **A** Relationships between functional diversity and sediment depths. **B** Relationships between taxonomical diversity and sediment depths. **C** Heatmaps of *Z*-score normalized relative abundances of key genes involved in CH_4_/N/S cycling. Blue dots/lines indicate the overall functional/taxonomical diversity of microbial communities; yellow dots/lines indicate the functional/taxonomical diversity of methane cycling communities; green dots/lines indicate the functional/taxonomical diversity of nitrogen cycling communities; purple dots/lines indicate the functional/taxonomical diversity of sulphur cycling communities. Grey shaded areas represent 95% confidence intervals. R^2^ was obtained by linear regression analysis and *P* was obtained by Pearson’s correlation analysis. *(0.01 < *P* < 0.05), **(0.001 < *P* < 0.01) and ***(*P* < 0.001)

To survey metagenomic characteristics along the sediment depth, we analysed the functional and taxonomical profiles based on metagenome sequencing data. We found distinct variation trends of functional and taxonomical diversity along the depth. Specifically, the overall functional α-diversity (based on KO terms) of microbial communities decreased with increasing depths (Fig. 2A). The functional diversity of N and S cycling communities deceased as sediment depths increased, while that of CH_4_ cycling communities did not show significant differences among depths (Fig. 2A). However, the overall taxonomical α-diversity of microbial communities as well as CH_4_, N and S cycling communities all increased with the sediment depth (Fig. 2B). Most of bacterial metagenome reads were matched to *Proteobacteria* (58.0–68.0%), and their relative abundances decreased significantly (R^2^ = 0.63, *P* < 0.001) along the sediment depth. Archaeal metagenome reads were mostly assigned to *Euryarchaeota* (0.8–2.3%), which showed a significantly (R^2^=0.19, *P* < 0.05) increasing trend along the sediment depth (Additional file 1: Fig. S2). Additionally, both functional and taxonomical profile-based principal coordinates analysis (PCoA) revealed that the sediment microbial community composition and structure significantly (*P* < 0.001, ANOSIM) shifted with the sediment depth (Additional file 1: Fig. S3, Fig. S4), suggesting that CH_4_, N and S cycling microbiomes were sensitive to the sediment depth.

### Vertical profiles of CH_4_/N/S cycling genes/pathways and taxa

To further understand the vertical distribution of functional genes and pathways of CH_4_, N and S cycling microbiomes, we analysed the functional composition of microbial communities with a focus on the relative abundance of key functional genes and pathways involved in CH_4_, N and S cycling (Fig. 2C), and a notable divergence of these metabolic patterns among different sediment depths was observed.

**Fig. 3:**
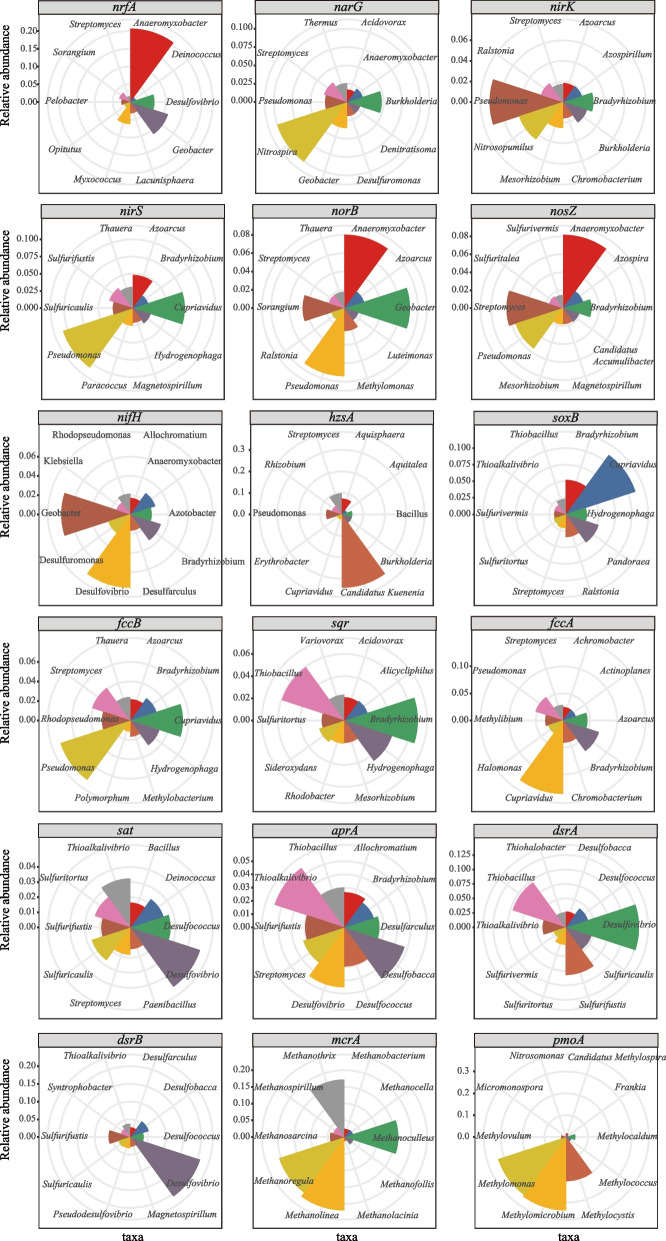
A summary of key microbial taxa involved in CH_4_, N and S cycling. The percentage of relative abundances for the top 10 abundant microbes involved in each key gene family is shown in the wind rose diagrams

(i) *CH*_*4*_*cycling*. We estimated the relative abundance of functional genes related to methanogenesis and methane oxidation. Our data showed a high percentage of metagenome reads matched central methanogenic pathways (21.8–28.6%) (Additional file 1: Fig. S5). The relative abundance of methyl coenzyme M reductase gene (*mcrA*) increased along the depth, whereas low frequencies of *pmoA* gene families were detected with a reverse trend (Fig. 2C, Additional file 1: Fig. S6). The main taxa responsible for methanogenesis were found to be *Methanolinea* (hydrogenotrophic methanogens), *Methanoregula* (hydrogenotrophic methanogens) and *Methanothrix* (acetoclastic methanogens). CH_4_ oxidizing bacteria were rarely obtained, appearing to be predominated by *Methylomicrobium*, *Methylomonas* and *Methylocystis* (Fig. 3). These results indicated relatively high abundances of gene families involved in methanogenesis were distributed in deep sediments, and methane production was mainly performed by hydrogenotrophic and acetoclastic methanogens.
(ii) *N cycling.* The metagenome reads mapped to the N cycle were mainly related to nitrate reduction with 15.4–16.5% for DNRA, 11.6–14.0% for denitrification, and 64.6–68.0% for organic N degradation and synthesis (Additional file 1: Fig. S5). First, gene families (*ureABC* and *gdh*) involved in organic N decomposition significantly (*P* < 0.05) decreased along the depth, which may explain high concentrations of NH_4_^+^ in the surface sediment to some extent. Second, all gene families involved in denitrification (*narG*, *nirK*, *nirS*, *norB*) were detected with high relative abundances but with a decreased trend along the sediment depth except *nosZ* (Fig. 2C, Additional file 1: Fig. S7), and the major denitrifying taxa included *Pseudomonas*, *Cupriavidus*, *Azoarcus*, *Sulfurifustis* and *Thauera* (Fig. 3, Additional file 1: Fig. S8). Third, the detected *nrfA* gene (the marker gene of DNRA) was largely affiliated with *Anaeromyxobacter*, *Geobacter* and *Desulfovibrio*, and their relative abundances decreased along the depth, whereas *nifH* gene originated from *Desulfovibrio*, *Geobacter* and *Bradyrhizobium* did not show differences among different sediment depths (Figs. 2C and 3, Additional file 1: Fig. S7). Fourth, the relative abundances of anammox genes (e.g. *hzo*, *hzsA*) were low, and a small number of metagenome reads were assigned to ammonia oxidizers. The results indicated that ammonification and nitrate reduction showed high functional potentials in the surface sediment (0–15 cm), and taxonomic groups responsible for nitrate reduction (denitrification and DNRA) were related to S-oxidizing bacteria and SO_4_^2−^-reducing bacteria.
(iii) *S cycling*. For the S cycle, the metagenome reads were largely mapped to organic S transformation (38.8–42.7%) and transformation between inorganic S and organic S (15.9–21.4%) (Additional file 1: Fig. S5). S oxidation genes (e.g. *fccAB* and *sqr* for sulphide oxidation, *soxB* for thiosulphate oxidation to sulphate) significantly (*P* < 0.05) decreased as depth increased, suggesting active S oxidation potentials in the surface sediment (Fig. 2C, Additional file 1: Fig. S9). These key S oxidation genes (*soxB*, *fccB* and *sqr*) were mostly affiliated with *Cupriavidus*, *Bradyrhizobium* and *Hydrogenophaga* (Fig. 3, Additional file 1: Fig. S8), pointing to the important role of these genera in S oxidation in mangrove sediments. Also, the relative abundance of gene families responsible for polysulphide reduction (*psrB*) and sulphite reduction (*asrB*) significantly (*P* < 0.05) increased along the depth (Fig. 2C, Additional file 1: Fig. S9), which were mostly affiliated with *Geobacter*, *Pseudomonas*, *Streptomyces* and *Desulfovibrio*. Additionally, the relative abundance of *sat* and *aprAB* for dissimilatory sulphate reduction increased at the depth of 0–30 cm and then decreased, while *dsrAB* exhibited no differences along the depth, which were affiliated with S-oxidizing bacteria (SOB) (dominated by *Thioalkalivibrio*, *Sulfurifustis*, *Sulfuricaulis* and *Thiobacillus*) and sulphate-reducing bacteria (SRB) (dominated by *Desulfovibrio*, *Desulfobacca* and *Desulfococcus*) (Figs. 2C and 3, Additional file 1: Fig. S9). The results indicated that S oxidation had high functional potentials in the surface sediment (0–15 cm), and sulphate reduction mainly occurred at the depth of around 30 cm. In view of taxonomical groups involved in denitrification and S oxidation, it is noted that S-driven denitrfiers, such as *Pseudomonas*, *Cupriavidus* and *Sulfurifustis*, could play an important role in coupling these two processes in the surface (0–15 cm) mangrove sediments.

### Microbial co-occurrence networks in mangrove sediments

As key gene families involved in CH_4_, N and S cycling showed distinct variation trends along the sediment depth, close correlations were detected among those processes. For example, gene families related to denitrification (*narG*, *nirK*, *nirS*, *norB* and *norC*) were significantly (*P* < 0.05) and positively correlated with S oxidation genes (*soxB*, *sqr* and *fccA*), suggesting a close relationship between these two processes (Additional file 1: Fig. S10). Also, significant (*P* < 0.05) and positive correlations were observed between *mcrABCD* gene families and *narG/nirK/nirS/norC/nosZ/asrB* gene families (Additional file 1: Fig. S10), indicating methanogenesis could be coupled with denitrification and sulphite reduction.

**Fig. 4:**
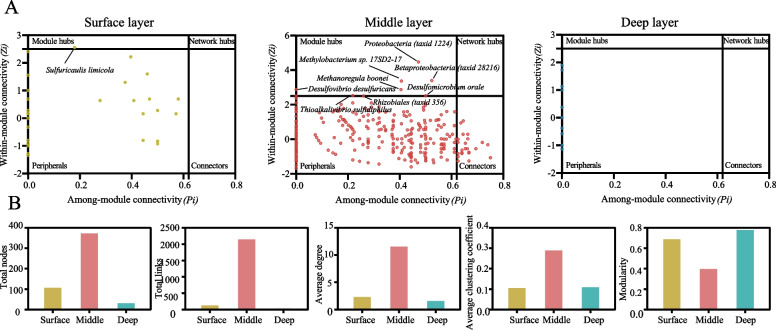
The co-occurrence networks of microbial communities in the surface layer (yellow), middle layer (red) and deep layer (blue) sediments. **A** The network nodes were separated by within-module connectivity (*Z*_*i*_) and among-module connectivity (*P*_*i*_). Threshold values of *Z*_*i*_ and *P*_*i*_ for categorizing microbial taxa were 2.5 and 0.62, respectively. **B** The overall topological characteristics at different sediment layers

To further explore possible microbial interactions and coupling mechanisms at different depths, we constructed co-occurrence networks using taxonomical profiles associated with all contigs involved in CH_4_, N and S cycling. Distinct structural and topological characteristics of CH_4_, N and S cycling microbiome networks were observed (Fig. 4B). The mid-layer network appeared to possess the largest complexity with more nodes, more links, higher average clustering coefficient, higher node degrees and lower modularity compared with the surface and deep layer networks. There were 80.8% positive associations and 19.2% negative associations in the mid-layer, while 76.0% positive and 24.0% negative associations in the surface layer, and 37.5% positive and 62.5% negative associations in the deep layer (Additional file 1: Fig. S11).

We also identified potential keystone species, including module hubs, network hubs and connectors based on within-module connectivity and among-module connectivity values. Only one module hub (*Sulfuricaulis limicola*) was found in the surface layer network, but the mid-layer network had eight module hubs: *Betaproteobacteria taxid* (28216), *Desulfomicrobium orale* DSM, *Desulfovibrio desulfuricans*, *Methanoregula boonei*, *Methylobacterium* sp. 17SD2-17, *Proteobacteria taxid* (1224), *Rhizobiales taxid* (356) and *Thioalkalivibrio sulfidiphilus* HL-EbGr7 (Fig. 4A). These potential keystone species were positively interacted with other microbial groups and could form microbial syntrophies in the middle mangrove sediments. Also, sulphate-reducing bacterium *Desulfovibrio desulfuricans* appeared to positively interact with methanogens (*Methanoculleus*, *Methanofollis* and *Methanolinea*), and *Methanoregula boonei* was positively correlated with *Desulfovibrio gigas* DSM in the middle layer, while those methanogens also had the maximal betweenness with other methanogens in the deep layer (Additional file 1: Table S6, Table S7), which mainly appeared to be hydrogenotrophic and acetoclastic methanogens. The results indicated that methanogens could potentially develop close relationships with SRB in the middle layer and with other methanogens in the deep layer.

### Genomic capability of coupling CH_4_, N and S cycling processes

Through metagenome assembly and binning, we further reconstructed 19 archaeal metagenome-assembled genomes (MAGs) and 58 bacterial MAGs (Additional file 2: Dataset S1). Among the archaeal MAGs, we retrieved five MAGs affiliated with *Methanoregulaceae*, *Methanoperedenaceae* and *Methanotrichaceae*, and they mainly distributed in deep sediment (Additional file 1: Fig. S12). *Methanoperedens* appeared to be an ANME with the marker gene (*mcrA*) for AOM, namely KO6.bin.60. Among the bacterial MAGs, we retrieved nine S-driven denitrifier MAGs containing key genes for S oxidation and denitrification, and nine MAGs for putative SRB with *dsrABD* genes or affiliated with *Desulfobacterota*. Only one MAG (KO6.bin.24) from the phylum *Desulfobacterota* encoded *dsrD* (Additional file 1: Fig. S13A, Fig. S14, Additional file 2: Dataset S2).

**Fig. 5:**
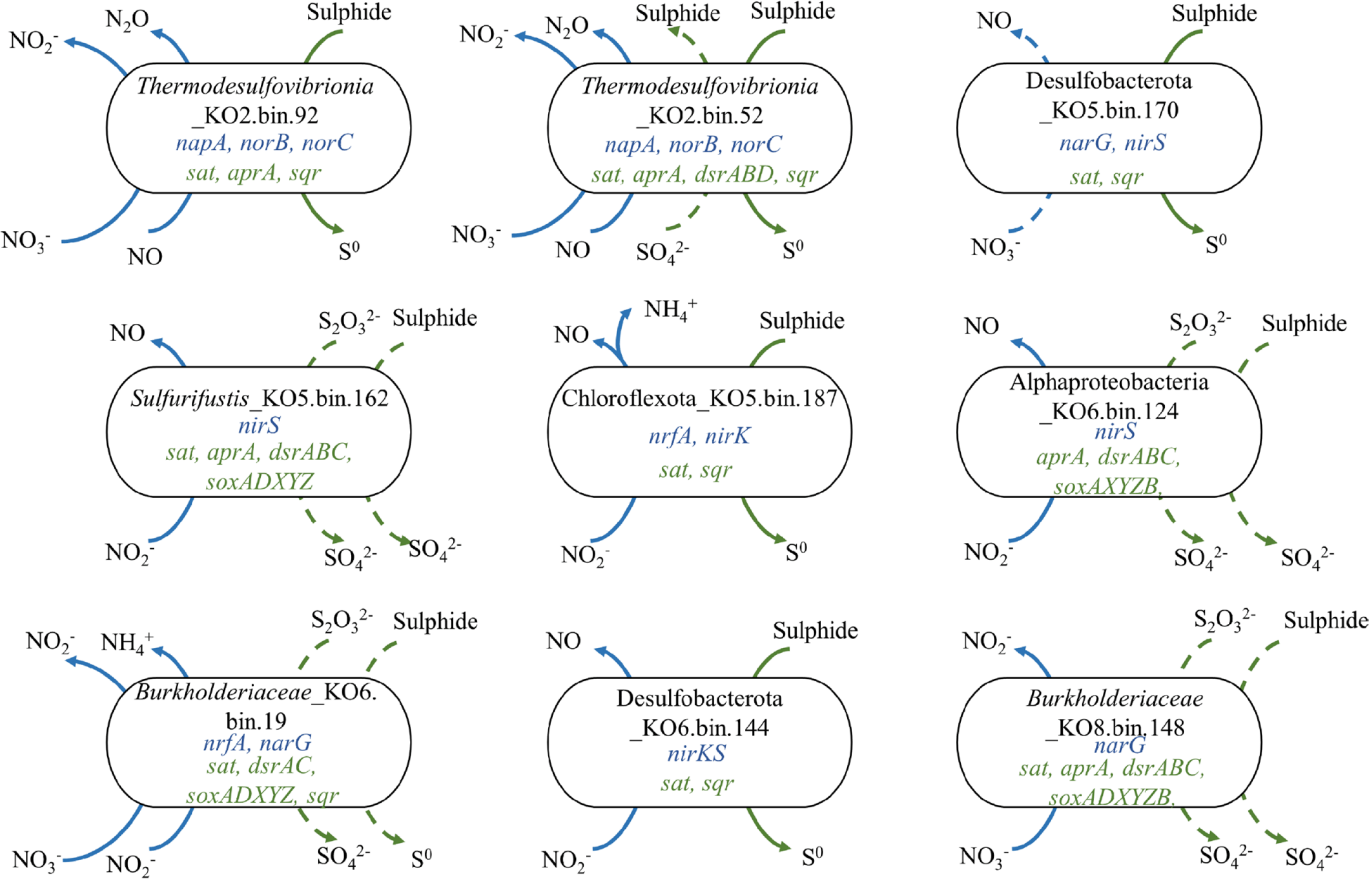
Metabolic characteristics of nine S-driven denitrifiers on denitrification and S oxidation in mangrove sediments. Blue arrows indicate denitrification reactions and green arrows indicate S oxidation reactions. Solid arrows represent the reactions found in the recovered MAGs and dashed arrows indicate omitted reactions. A detailed list of genes in these S-driven denitrifiers can be found in Additional file 2: Dataset S3

S-driven denitrifier MAGs were distributed throughout *Thermodesulfovibrionia* (KO2.bin.92, KO2.bin.52), *Desulfobacterota* (KO5.bin.170, KO6.bin.144), *Sulfurifustis* (KO5.bin.162), *Chloroflexota* (KO5.bin.187), *Alphaproteobacteria* (KO6.bin.124) and *Burkholderiales* (KO6.bin.19, KO8.bin.148) (Fig. 5). The relative abundances of these MAGs were higher in the surface and middle sediments than those in the deep sediment (Additional file 1: Fig. S13B). All S-driven denitrifier MAGs could use different N species as electron acceptors except N_2_O, which could lead to N_2_O production in the sediment. Among them, six MAGs harboured sulphide:quinone oxidoreductase (Sqr), which catalyses the oxidation of sulphide to elemental sulphur (Fig. 5).

**Fig. 6:**
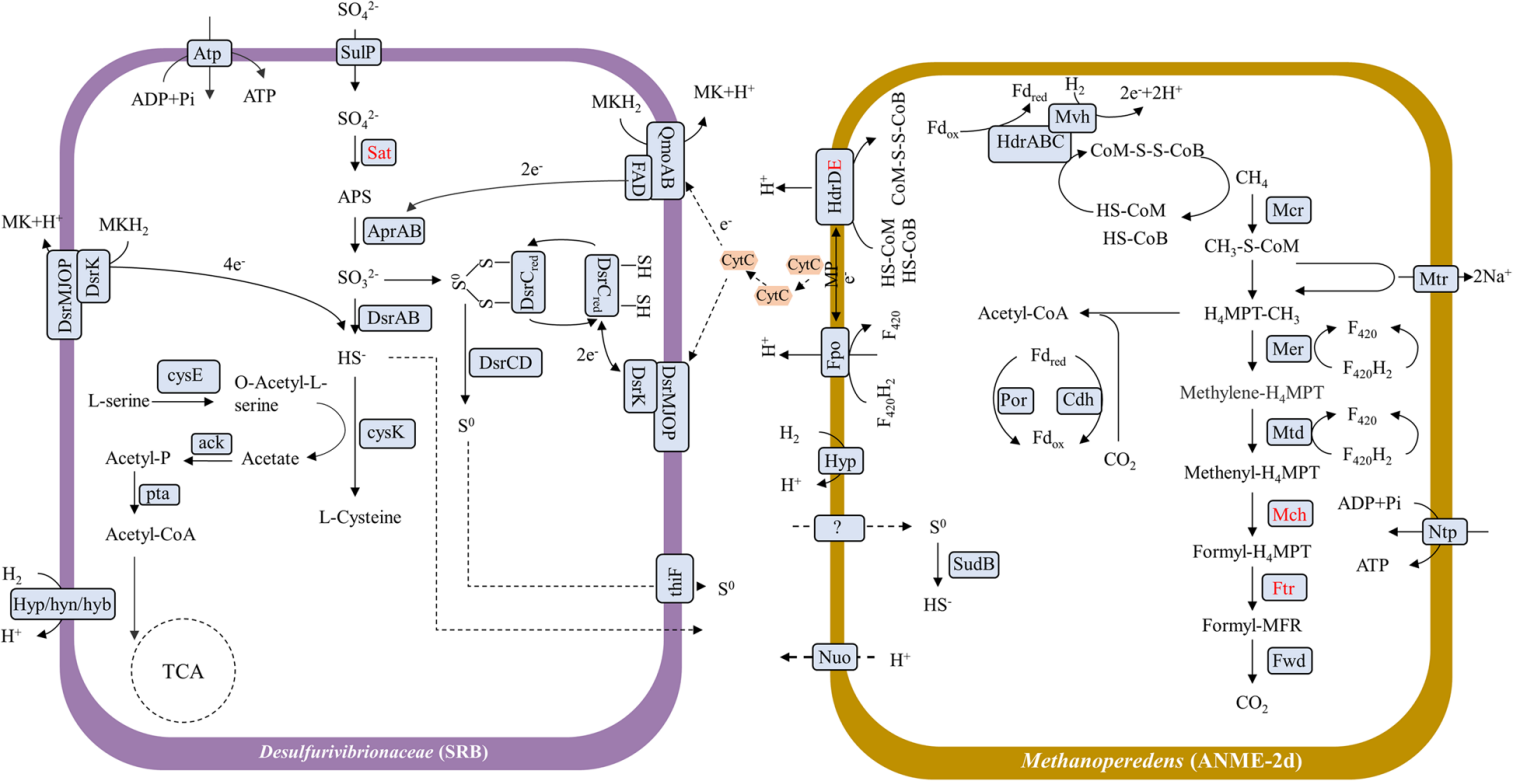
Possible coupling pathways of anaerobic oxidation of methane and sulphate reduction inferred from the MAGs of *Methanoperedenaceae* and *Desulfurivibrionaceae*. Absent enzymes were in red colour; dashed arrows indicate unknown pathways. The detailed list of genes in these genomes can be found in Additional file 2: Dataset S3 and Dataset S4

As a representative genome for SRB, *Desulfurivibrionaceae* (KO6.bin.24) contained core enzymes involved in SO_4_^2−^ reduction, including adenylylsulphate reductase alpha subunits (AprA), and dissimilatory sulfitereductase alpha, beta and delta subunits (DsrABD) except sulphate adenylyltransferase (Sat). It also contained electron transport complexes QmoAB and DsrMKJOP, and the sulphur relay protein DsrC. Additionally, a sulphur transporter (ThiF) was also detected in this *Desulfurivibrionaceae* genome (Fig. 6, Additional file 2: Dataset S3).

As a representative ANME, *Methanoperedens* (KO6.bin.60) contained core enzymes involved in AOM except methenyl-H_4_MPT cyclohydrolase (Mch) and formyl-MFR:H_4_MPT formyltransferase (Ftr): formyl-MFR dehydrogenase (Fmd), methylene-H_4_MPT dehydrogenase (Mtd), methylene-H_4_MPT reductase (Mer), methyl-H_4_MPT:HS-CoM methyltransferase (Mtr) and methyl-CoM reductase (Mcr). It also contained the heterodisulfide reductase (Hdr), F_420_ hydrogenases (Fpo) and some extracellular electron transfer proteins, such as archaeal type IV pilus (PilTC) and archaeal flagellin (FlaD). Additionally, sulphur-related enzymes were detected in this genome, such as sulphur transfer proteins (TauE) and sulphide dehydrogenase (SudB), which could reduce S^0^ to sulphide (Fig. 6, Additional file 2: Dataset S4).

## Discussion

Revealing the vertical profile of CH_4_, N and S cycling genes/pathways and associated taxonomic groups as well as their potential coupling mechanisms is crucial to understand mangrove ecosystem functioning. In this study, we analysed the metabolic landscape of CH_4_, N and S cycling microbial communities to decipher potential interactions among functional members. Our results clearly demonstrated a decreased trend of the overall functional diversity of microbial communities along the sediment depth, and a strong vertical stratification of microbial functional genes/pathways was predominantly driven by AVS and pH, especially high abundances of key gene families involved in denitrification and S oxidation distributing in the surface layer and relatively high abundances of key gene families involved in methanogenesis distributing in the deep layer. Complex microbial interactions were found in the surface and middle layers with S oxidation coupled with denitrification in the surface sediment, and SRB with methanogens/ANMEs in the middle layer. These results generally support our core hypothesis that fluctuating environmental conditions would lead to a notable divergence of metabolic patterns of CH_4_/N/S cycling, and S cycling microbes could play important roles in coupling CH_4_ and N cycling processes in mangrove sediments.

CH_4_ cycling is an important process of the C cycle, and its emissions significantly offset the C burial in mangrove sediments, accelerating global warming [65]. Previous studies showed that the abundance of methanogenesis genes increased with the mudflat sediment depth [66], and the composition of methanogens showed obvious variations in different ecosystems [6, 67]. For example, hydrogenotrophic methanogenesis was dominated in a subseafloor sediment [68], peatlands [69] and mangrove sediments [70]. In this study, we observed a relatively high abundance of *mcrA* gene and an increasing trend along the sediment depth, suggesting a possibly more active methanogenesis in the deep sediment. Also, *Methanolinea* and *Methanoregula* of the order *Methanomicrobiales* were found to be the dominant methanogenic genera. This finding is consistent with previous studies in the Sanjiang Mire Wetland, Ruoergai peatland, Hongze wetland and Poyang wetland [71], indicating that hydrogenotrophic methanogenesis may dominate CH_4_ production in mangrove sediments. Specifically, *Methanoregula boonei* as a H_2_-utilizing methanogen was identified as a keystone in the middle mangrove sediment, and it also had the maximum betweenness with other methanogens in the deep sediment. As *M. boonei* generated ATP using protons with membrane-bound methyltransferase rather than sodium ions, hydrogen might be transferred from other methanogens to *M. boonei* to conserve energy with close interactions between them [72, 73]. Therefore, the close interactions among methanogens in deep sediments might contribute to the CH_4_ generation and regulating CH_4_ emissions in mangrove sediments [74]. In addition, low abundances of *pmoA* gene were detected and their abundances decreased with the sediment depth, which was consistent with previous studies [75, 76].

Microbially driven N cycling is critical to maintain N nutrients for mangrove growth in such N-limited ecosystems. Denitrification is considered as the major N-loss pathway in mangrove sediments through the production of N_2_O (a strong greenhouse gas) and/or N_2_ [9], while another N-loss pathway, anammox, was found to mainly occur in shallow wetland soils associated with high concentrations of NH_4_^+^ as substrate in previous studies [14, 77]. In contrast to anammox and denitrification, dissimilatory nitrate reduction to ammonium (DNRA) reduces nitrate to NH_4_^+^ and was found to alleviate N limitation of mangrove ecosystems [78]. Our results showed higher functional potentials of denitrification in the surface sediment compared to the deep sediment, consistently with previous studies [16], which could be explained by two possible mechanisms: (i) co-respiration of NO_x_^−^ and O_2_ [17] and (ii) closely coupled nitrification–denitrification in microenvironments [79]. Therefore, our results implied that some denitrifying bacteria could adapt to tidally induced recurrent redox oscillations and/or tolerate certain concentrations of O_2_ and perform denitrification in the surface sediment. Differently from previous studies, we found an increasing trend of anammox along the sediment depth, mainly due to competition for inorganic N compounds by growing plants [80] or heterotrophic nitrate- and nitrite-reducing bacteria. In addition, due to the reduction of root density along mangrove sediments [81], the N demand for mangrove growth decreased. High NH_4_^+^ concentration, organic N decomposition and DNRA potential were observed in the surface sediment, suggesting high demands of N supply in the surface sediment, and these two processes may contribute to an important N sink in the surface mangrove sediment [82, 83].

For S metabolism, mangroves under macro-tidal regimes are rich in sulphate, driving efficient S cycling. Sulphate reduction is considered as the most important respiration process in mangrove sediments. The abundance of sulphate-reducing microorganisms is usually low in the uppermost oxygenated layers of sediments, while it reaches a maximum in the underlying anoxic zones and then decreases into the sulphate-depleted CH_4_ zone [84, 85]. Due to the abundant sulphide produced by sulphate reduction in mangrove sediments, S oxidation may benefit plants by removing potentially toxic sulphide from the root zone [86]. Consistently with previous studies, a similar trend of gene families involved in sulphate reduction (*sat*, *aprA*, *aprB*) was observed in our study. However, the relative abundance of *dsrA* or *dsrB* did not show significant differences along the sediment depth, probably because both genes are also involved in multi-step processes of S oxidation as a reverse reaction for sulphate reduction [87]. Additionally, a high functional potential of S oxidation mainly performed by *Cupriavidus*, *Bradyrhizobium* and *Hydrogenophagain* was observed in the surface sediment in our study, where oxygen from recurrent tides and released by mangrove roots could provide suitable redox conditions for S oxidation [86]. Together, our study highlights that S oxidation and sulphate reduction may play an important ecological role in the surface and middle layer sediments, respectively, and high potentials of S metabolism may guarantee suitable conditions for mangrove growth.

Through developing diverse syntrophic partnerships, microorganisms could overcome thermodynamic barriers imposed by the environment to maintain their metabolic activities [88]. The co-occurrence networks constructed in this study showed potential microbial interactions of functional groups involved in CH_4_, N and S cycling. Generally, a more complex network was found in the middle layer sediment as microbial interactions could be reduced due to heterogeneous environments in the surface sediment and excessive environmental pressures in the deep sediment [27]. Therefore, various syntrophic consortia could be responsible for diverse coupling mechanisms in different sediment depths.

It was proposed that denitrification coupled with S oxidation was crucial for the detoxification of sulphide in the mangrove sediment [19], which could be performed by a single autotrophic microbe such as *α-*, *β-*, *γ-* and *ε-proteobacteria* or by cooperation among those microorganisms [28, 89], indicating a diverse range of potential overlaps between denitrification and S oxidation. Previous studies showed that S-driven autotrophic denitrification coupling with the oxidation of reduced inorganic S compounds was widely distributed in the marine sediment [90] and soda lakes [91]. In this study, we identified a keystone SOB (*Sulfuricaulis limicola*) in the surface sediment, and observed strong correlations among gene families involved in S oxidation and denitrification as well except *nosZ* gene, suggesting a potential coupling mechanism of S oxidation with denitrification. As a N_2_O sink, N_2_O flux in mangrove sediments has ever been estimated ranging from −0.2 to 6.3 mg m^−2^ day^−1^ [92, 93], and denitrification could be responsible for up to 43 to 93% of N_2_O production [94], indicating that denitrifiers play an important role in N_2_O production. Diverse S-driven denitrifiers have been isolated from mangrove sediments, such as the centimetre-long bacterium *Candidatus (Ca.) Thiomargarita magnifica*, which could oxidize hydrogen sulphide and reduces nitrate, with ~75% of its total cell volume being occupied by a large nitrate-containing vacuole [95, 96]. Also, both read- and genome-level analyses found that *Cupriavidus* affiliated with family *Burkholderiaceae* and *Sulfurifustis* had the capability of S-oxidizing denitrification, which were found to be important contributors to both S oxidation and denitrification [97–99]. These findings suggest a more tremendous diversity of S-oxidizing denitrifier lineages in mangrove sediments than previously thought. Furthermore, previous studies found that high sulphide concentrations could inhibit nitrous oxide (N_2_O) reductases [100, 101], and soil N–S interactions could substantially influence N_2_O emissions [102]. In this study, all S-driven denitrifier MAGs lacked the *nosZ* gene, suggesting that such sulphide-utilizing groups might be an important contributor to N_2_O production in the surface mangrove sediment [97]. Overall, the S-driven denitrifier-mediated N-S coupling reaction proceeds in the direction that is conductive to the sulphide detoxification, thus elucidating the metabolic mechanism of S-driven denitrification could regulate greenhouse gas emissions in the mangrove sediment.

The relationship of SRB and methanogens has been extensively studied in various environments such as marine sediments [103], salt marsh sediments [104] and mangrove sediments [105]. Although SRB were known to outcompete methanogens over common substrates, such as acetate and H_2_, channeling the electron flow towards CO_2_ production rather than methane [106], several studies showed that SRB and methanogens could co-exist under high sulphate concentrations, especially in the estuarine sediment [105, 107, 108]. In this study, we noticed that methanogens and SRB were keystones and positively correlated with each other, suggesting that they might co-exist in mangrove sediments. Although there are thermodynamic barriers of this interaction, we speculate that the co-occurrence between methanogens and SRB can be pulled forward upon the demand of ANME and SOB with recycling of metabolic products among them [109]. Enrichment experiments with deep groundwater indicated *Methanoperedenaceae* (ANME-2d) could conduct sulphate-dependent anaerobic oxidation of methane in the terrestrial subsurface [110]. Similarly, a *Methanoperedenaceae* MAG retrieved in this study had the metabolic potential for extracellular electron transfer to a syntrophic partner, such as flagella, which was previously reported to be an important feature of syntrophy establishments [111]. A previous study provided the genomic evidence that *Methanoperedenaceae* could acquire sulphur reduction genes by lateral gene transfer and might be involved in respiratory sulphur-dependent AOM [112]. The *Methanoperedenaceae* in this study also could encode sulphide dehydrogenase and reduce S^0^, where the S^0^ could form by SRB and be exported across the cytoplasmic membrane by ThiF protein, a sulphur exporter [113]. Also, methanotrophic archaea were found to produce S^0^ during AOM and the produced S^0^ would be disproportionated by the *Deltaproteobacteria* in a previous study [31], while our results suggest other possible mechanisms that *Methanoperedens* might directly transfer electrons to *Desulfurivibrionaceae*, or by AOM *Methanoperedens* could ultilize S^0^ as an electron acceptor, which could be produced by *Desulfurivibrionaceae*. Recently, synthetic microbial ecology methods are considered as a novel approach to verify microbial interactions among different microorganisms like niche differentiation among different types of nitrifiers [114], but an experimental verification of those potential syntrophic relationships is a great challenge due to the difficulty for isolating or culturing ANME or similar microorganisms.

As mangroves are located at the transition between ocean and land, the vertical variation of environmental factors caused by periodically tidal fluctuations play important roles in shaping the mangrove sediment microbiome structure [15]. Several previous studies showed that pH had a strong filtering impact on microbial communities [115, 116]. For instance, methanogens were sensitive to pH, which may affect acetate availability and directly impact acetoclastic methanogenesis, resulting in increased abundances of methanogens along the sediment depth [66, 117, 118]. Also, the availability of electron acceptors and donors could influence energetic metabolisms and further impact microbial functions [21, 22]. For example, the availability of electron acceptors (SO_4_^2−^, NO_3_^−^/NO_2_^−^, Mn_5_^+^) had a strong selective effect on ANMEs and then on CH_4_ oxidation [119]. In this study, we found sediment niches of microbial communities differentiated from surface to deep layers, which was largely driven by various environmental factors including AVS and pH. First, AVS was detected as an important substrate for S oxidation and sulphate reduction in mangrove sediments, which could couple with nitrate reduction and methanogenesis, respectively, and the surface sediment with high concentrations of AVS showed high functional potentials of S oxidation, suggesting its important role in driving element cycles and sulphide detoxification for mangroves [10]. Second, increased pH could increase the relative abundance of *mcrA* gene in the deep sediment [120, 121], while neutral pH conditions might be suitable for methanogen growth [122]. Third, our study found that metabolic pathways of nitrate reduction were mainly driven by pH and AVS. This may be because that pH could directly affect the activity of enzymes involved in nitrate reduction and indirectly affect nitrate reduction by regulating the availability of organic C for associated microorganisms with AVS as an important electron donor [123]. Together, our study indicated that environmental factors especially pH and AVS made great contributions to shaping the vertically stratified CH_4_, N, and S cycling processes, among which, AVS was a critical electron donor impacting mangrove sediment S oxidation and denitrification. High abundances of gene families involved in S oxidation and denitrification were distributed in the surface sediment and coupled by S-oxidizing denitrifers, while SRB could flexibly interact with methanogens possibly by AOM in the middle layer, indicating the important role of S cycling microbes in microbial interactions and functions in mangrove ecosystems.

## Conclusions

**Fig. 7:**
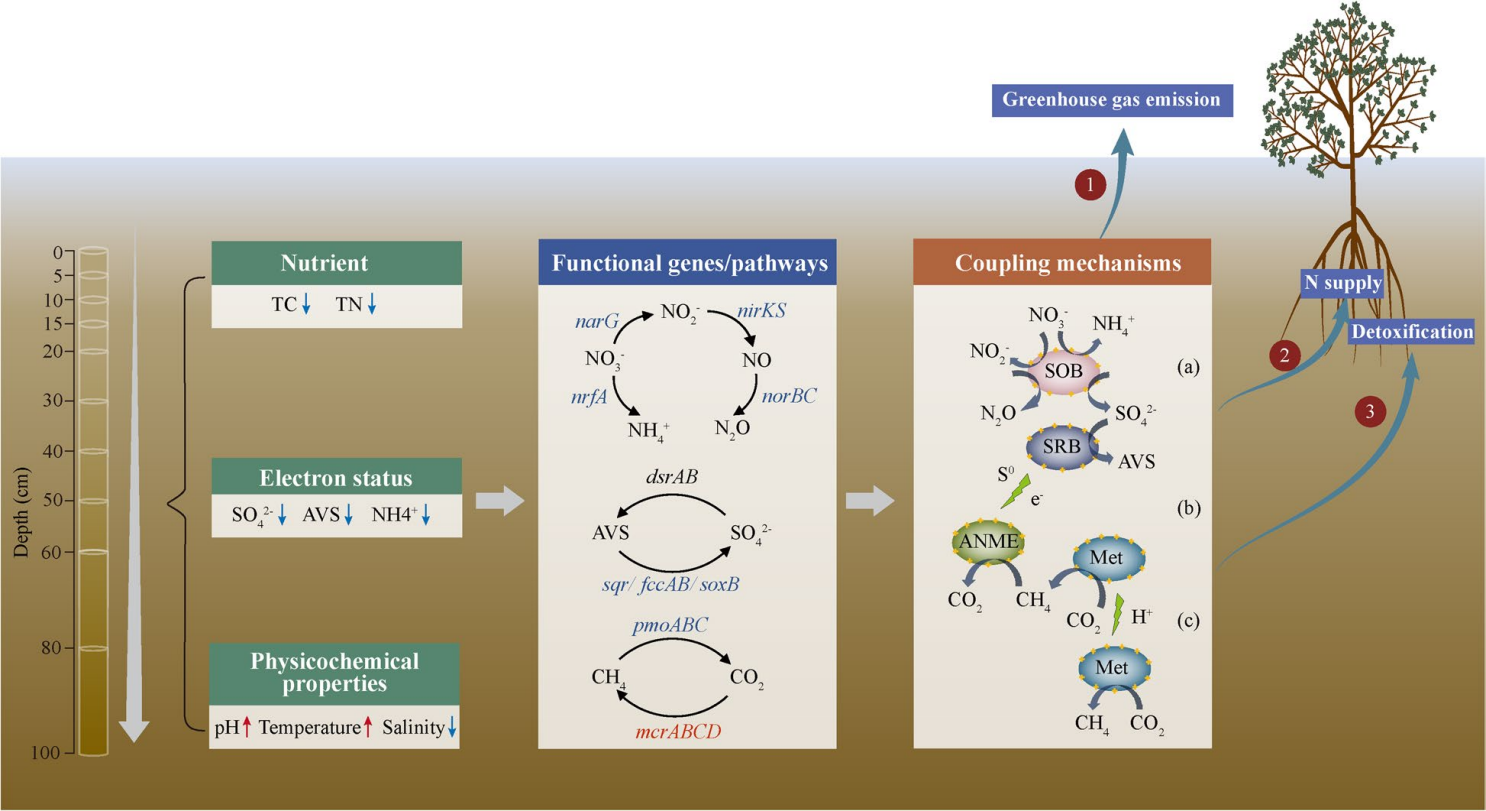
A conceptual model of depth-related microbially driven CH_4_, N and S cycling and their coupling mechanisms in the mangrove sediment. First, physicochemical properties differed vertically by depths and these changes, especially AVS and pH, had a great effect on the distribution of functional genes/pathways involved in CH_4_, N and S cycling. Second, possible coupling mechanisms were proposed: a SOB-denitrifier/DNRA; b SRB-methanogen; c Methanogen-methanogen. The relative abundance of functional genes involved in denitrification and S oxidation was enriched in the surface sediment and they could be coupled by S-driven autotrophic denitrifiers, such as *Burkholderiaceae* and *Sulfurifustis*, whereas those involved in dissimilatory sulphate reduction and methanogenesis were enriched in the middle and deep sediments. SRB could co-exist with methanogens in the middle sediment, which could be pulled forward upon the demand of ANME and SOB or directly transfer H^+^ or electrons. Third, the depth-related CH_4_, N and S cycling microbiomes and their coupling processes may have a great effect on mangrove ecosystem functions and services, such as greenhouse gas emissions, N supply for plant growth, and detoxification. The red and blue colours of words and arrows represent increase or decrease of environmental variables or key genes, respectively along the sediment depth. Black-coloured genes indicate no momentous differences along the sediment depth. Yellow arrows represent H^+^/e^−^ transfer between microbes. TC: total carbon; TN: total nitrogen; AVS: acid volatile sulphide; SOB: sulphur oxidizer; SRB: sulphate reducer; ANME: anaerobic methane oxidizer; Met: methanogen

Our metagenome sequencing analysis unveiled vertically stratified CH_4_, N and S cycling microbiomes and their coupling mechanisms in the mangrove sediment (Fig. 7). The results showed distinct variations of CH_4_, N and S cycling genes/pathways mainly driven by pH and AVS along the sediment depth, and S cycling microbes could play key roles in coupling important biogeochemical processes, such as S oxidation and denitrification, methanogenesis and sulphate reduction, and anaerobic methane oxidation and sulphate reduction. S oxidation coupled with denitrification was found to be dominant in the surface sediment, while SRB might develop syntrophic relationships with ANME by direct electron transfer or zero-valent sulphur, and pull forward the co-existence of methanogens and SRB in the middle and deep sediments. The coupling of those biogeochemical processes may ensure the metabolic versatility of CH_4_, N and S cycling microbiomes, maintaining mangrove ecosystem functions and services, such as detoxification, N supply for plant growth and greenhouse gas emissions. This study provides new insights into a comprehensive understanding of microbially driven CH_4_, N, and S cycling genes/pathways and possible coupling mechanisms along the sediment depth in mangrove ecosystems. Future studies are needed to clarify ecological and molecular mechanisms of energy and matter transfers involved in each of metabolic pathways to depict a full picture of tightly coupled biogeochemical processes in mangrove sediments.

## Supplementary Information

**Additional file 1: Table S1.** Summary of sequence information of different samples in each step of metagenome sequencing analysis. **Table S2.** Multiple response permutation procedure (MRPP) analysis of functional profiles related to methane, nitrogen and sulphur cycling among different depths. **Table S3.** Analysis of similarity (ANOSIM) based on functional profiles related to methane, nitrogen and sulphur cycling among different depths. **Table S4.** The vertical distribution of physicochemical characteristics of all mangrove sediment samples in this study. **Table S5.** Summary statistics for Mantel tests of correlations between metabolic pathways and environmental factors. **Table S6.** Summary of correlations between functional groups and keystones. **Table S7.** Summary of correlations between functional groups in the deep sediment. **Fig. S1.** The location of the sampling site at the Qi’ao Mangrove Reserve, Zhuhai, Guangdong province, China. **Fig. S2.** The depth-dependent profile of community structure in mangrove sediments (a) and linear regression analysis of correlations between the relative abundance of *Proteobacteria* and *Euryarchaeota* (b). **Fig. S3.** Principal coordinate analysis (PCoA) plot of all microbial functions (a), methane cycling (b), nitrogen cycling (c), and sulphur cycling (d). **Fig. S4.** Principal coordinate analysis (PCoA) plot of all microbial communities (a), and CH_4_-cycling (b), N-cycling (c) and S-cycling (d) microbial communities. **Fig. S5.** A heatmap plot of functional pathways for predicted open reading frames (ORFs) from metagenome sequence reads for sediment samples at different depths. **Fig. S6.** The vertical distribution of relative abundances of key gene families involved in methane cycling. **Fig. S7.** The vertical distribution of relative abundances of key gene families involved in nitrogen cycling. **Fig. S8.** The vertical distribution of the relative abundance of key microbial taxa (top 5) responsible for genes involved in S oxidation and denitrification. **Fig. S9.** The vertical distribution of relative abundances of key gene families involved in sulphur cycling. **Fig. S10.** Correlations between the relative abundance of key gene families. **Fig. S11.** Methane, nitrogen and sulphur cycling co-occurrence networks of three sediment layers at the species level with OTUs colored by phylum/class. **Fig. S12.** The vertical distribution of relative abundances of retrieved methanogen/ANME/SRB MAGs. **Fig. S13.** Metabolic profiles of retrieved MAGs (A) and relative abundances of selected MAGs (B). **Fig. S14.** Carbon, nitrogen, sulphur and metal metabolisms involved in different selected lineages.

**Additional file 2: Dataset S1.** Genomic information of high quality bins. **Dataset S2.** METABOLIC HMM hits in MAGs. **Dataset S3.** Desulfurivibrionaceae (KO6.bin.24) metabolism. "+" = gene present in the MAG. "Absent" = not present in the MAG. **Dataset S4.** Methanoperedens (KO6.bin.60) metabolism. "+" = gene present in the MAG. "Absent" = not present in the MAG.

## Data Availability

The metagenomic data of microbial communities in mangrove sediments were deposited in National Omics Data Encyclopedia (NODE) under accession number OEX012906. The authors declare that the primary data supporting the findings of this study are available within this article and in the additional files. Extra data supporting the findings of this study are available from the corresponding authors upon request.

## Abbreviations

ANME: Anaerobic CH_4_ oxidizer
ANOSIM: Analysis of similarity
ANOVA: Analysis of variance
AOM: Anaerobic oxidation of methane
AVS: Acid volatile sulphide
C: Carbon
CH_4_: Methane
MAG: Metagenome-assembled genome
MENA: Molecular ecological network analysis
MIC: Maximal information coefficient
MRPP: Multiple response permutation procedure
N: Nitrogen
ORF: Open reading frame
PCoA: Principal coordinates analysis
S: Sulphur
SDS: Sodium dodecyl sulphate
SOB: s-oxidizing bacteria
SRB: Sulphate-reducing bacteria
TC: Total carbon
TN: Total nitrogen
TPM: Transcripts per million
TS: Total sulphur
