# Supplementary material for: Vertically stratified methane, nitrogen and sulphur cycling and coupling mechanisms in mangrove sediment microbiomes
Source: Microbiome. 2023 Apr 5;11:71. doi: 10.1186/s40168-023-01501-5 (PMC10074775; doi:10.1186/s40168-023-01501-5)
Supplement: Supplementary file 2 — Additional file 1: Table S1. Summary of sequence information of different samples in each step of metagenome sequencing analysis. Table S2. Multiple response permutation procedure (MRPP) analysis of functional profiles related to methane, nitrogen and sulphur cycling among different depths. Table S3. Analysis of similarity (ANOSIM) based on functional profiles related to methane, nitrogen and sulphur cycling among different depths. Table S4. The vertical distribution of physicochemical characteristics of all mangrove sediment samples in this study. Table S5. Summary statistics for Mantel tests of correlations between metabolic pathways and environmental factors. Table S6. Summary of correlations between functional groups and keystones. Table S7. Summary of correlations between functional groups in the deep sediment. Fig. S1. The location of the sampling site at the Qi’ao Mangrove Reserve, Zhuhai, Guangdong province, China. Fig. S2. The depth-dependent profile of community structure in mangrove sediments (a) and linear regression analysis of correlations between the relative abundance of Proteobacteria and Euryarchaeota (b). Fig. S3. Principal coordinate analysis (PCoA) plot of all microbial functions (a), methane cycling (b), nitrogen cycling (c), and sulphur cycling (d). Fig. S4. Principal coordinate analysis (PCoA) plot of all microbial communities (a), and CH4-cycling (b), N-cycling (c) and S-cycling (d) microbial communities. Fig. S5. A heatmap plot of functional pathways for predicted open reading frames (ORFs) from metagenome sequence reads for sediment samples at different depths. Fig. S6. The vertical distribution of relative abundances of key gene families involved in methane cycling. Fig. S7. The vertical distribution of relative abundances of key gene families involved in nitrogen cycling. Fig. S8. The vertical distribution of the relative abundance of key microbial taxa (top 5) responsible for genes involved in S oxidation and denitrification. Fig. S9. T [file 40168_2023_1501_MOESM1_ESM.docx]

**SUPPORTING INFORMATION**

1. **Supplementary tables**

**Table S1** Summary of sequence information of different samples in each step of metagenome sequencing analysis

**Table S2** Multiple response permutation procedure (MRPP) analysis of functional profiles related to methane, nitrogen and sulphur cycling among different depths

**Table S3** Analysis of similarity (ANOSIM) based on functional profiles related to methane, nitrogen and sulphur cycling among different depths

**Table S4** The vertical distribution of physicochemical characteristics of all mangrove sediment samples in this study

**Table S5** Summary statistics for Mantel tests of correlations between metabolic pathways and environmental factors

**Table S6** Summary of correlations between functional groups and keystones

**Table S7** Summary of correlations between functional groups in the deep sediment

1. **Supplementary figures**

**Fig. S1** The location of the sampling site at the Qi’ao Mangrove Reserve, Zhuhai, Guangdong province, China

**Fig. S2** The depth-dependent profile of community structure in mangrove sediments (a) and linear regression analysis of correlations between the relative abundance of *Proteobacteria* and *Euryarchaeota* (b)

**Fig. S3** Principal coordinate analysis (PCoA) plot of all microbial functions (a), methane cycling (b), nitrogen cycling (c), and sulphur cycling (d)

**Fig. S4** Principal coordinate analysis (PCoA) plot of all microbial communities (a), and CH_4_-cycling (b), N-cycling (c) and S-cycling (d) microbial communities

**Fig. S5** A heatmap plot of functional pathways for predicted open reading frames (ORFs) from metagenome sequence reads for sediment samples at different depths

**Fig. S6** The vertical distribution of relative abundances of key gene families involved in methane cycling

**Fig. S7** The vertical distribution of relative abundances of key gene families involved in nitrogen cycling

**Fig. S8** The vertical distribution of the relative abundance of key microbial taxa (top 5) responsible for genes involved in S oxidation and denitrification

**Fig. S9** The vertical distribution of relative abundances of key gene families involved in sulphur cycling

**Fig. S10** Correlations between the relative abundance of key gene families

**Fig. S11** Methane, nitrogen and sulphur cycling co-occurrence networks of three sediment layers at the species level with OTUs colored by phylum/class

**Fig. S12** The vertical distribution of relative abundances of retrieved methanogen/ANME/SRB MAGs

**Fig. S13** Metabolic profiles of retrieved MAGs (A) and relative abundances of selected MAGs (B)

**Fig. S14** Carbon, nitrogen, sulphur and metal metabolisms involved in different selected lineages

1. **Supplementary tables**

**Table S1** Summary of sequence information of different samples in each major step of metagenome sequencing analysis.

| Sample | Raw Reads | Raw Base(G) | Q20 (%) | Q30 (%) | GC Content (%) | Qualified Reads | Merged Reads |
| --- | --- | --- | --- | --- | --- | --- | --- |
| ZH-Q0-5-1 | 39093129 | 11.73 | 96.88 | 91.98 | 59.99 | 34606417 | 17872173 |
| ZH-Q0-5-2 | 35789446 | 10.74 | 96.96 | 92.16 | 58.28 | 31838513 | 15375518 |
| ZH-Q0-5-3 | 40938057 | 12.28 | 96.75 | 91.47 | 59.79 | 36148697 | 7186994 |
| ZH-Q0-5-4 | 37674841 | 11.3 | 96.56 | 91.25 | 60.07 | 32883395 | 9544945 |
| ZH-Q0-5-5 | 34072469 | 10.22 | 97.01 | 92.03 | 57.66 | 30448662 | 9061589 |
| ZH-Q5-10-1 | 39632706 | 11.89 | 96.69 | 91.48 | 57.26 | 34846093 | 12363889 |
| ZH-Q5-10-2 | 37351666 | 11.21 | 96.72 | 91.56 | 57.49 | 32881517 | 11999254 |
| ZH-Q5-10-3 | 38292544 | 11.49 | 96.69 | 91.25 | 58.44 | 33765221 | 10517851 |
| ZH-Q5-10-4 | 45955254 | 13.79 | 96.67 | 91.49 | 62.6 | 40291210 | 14064158 |
| ZH-Q5-10-5 | 38986911 | 11.7 | 96.61 | 91.35 | 59.39 | 34126182 | 10019152 |
| ZH-Q10-15-1 | 47170312 | 14.15 | 97.01 | 92.15 | 61.63 | 42085161 | 16413492 |
| ZH-Q10-15-2 | 66546667 | 19.96 | 97.54 | 93.49 | 60.29 | 60544329 | 58865523 |
| ZH-Q10-15-3 | 40865433 | 12.26 | 96.89 | 91.88 | 59.18 | 36238772 | 12879577 |
| ZH-Q10-15-4 | 46556802 | 13.97 | 96.77 | 91.63 | 57.23 | 41088683 | 13946952 |
| ZH-Q10-15-5 | 39287230 | 11.79 | 96.7 | 91.58 | 59.3 | 34513120 | 13669507 |
| ZH-Q15-20-1 | 42921407 | 12.88 | 96.73 | 91.68 | 62.02 | 37717955 | 16731248 |
| ZH-Q15-20-2 | 44028520 | 13.21 | 96.89 | 91.91 | 57.65 | 39142844 | 10887026 |
| ZH-Q15-20-3 | 49419311 | 14.83 | 96.73 | 91.63 | 58.52 | 43563653 | 13621803 |
| ZH-Q15-20-4 | 40626725 | 12.19 | 96.49 | 91.16 | 59.73 | 35319288 | 11915506 |
| ZH-Q15-20-5 | 33504696 | 10.05 | 96.99 | 92.07 | 55.34 | 29899853 | 11914085 |
| ZH-Q20-30-1 | 35044724 | 10.51 | 96.69 | 91.47 | 59.42 | 30862340 | 6687057 |
| ZH-Q20-30-2 | 46247908 | 13.87 | 96.74 | 91.62 | 57.09 | 40749313 | 15278919 |
| ZH-Q20-30-3 | 67951834 | 20.39 | 96.7 | 91.53 | 59.15 | 59788364 | 18781442 |
| ZH-Q20-30-4 | 39541019 | 11.86 | 97.94 | 94.16 | 56.26 | 36818944 | 24346382 |
| ZH-Q20-30-5 | 61093695 | 18.33 | 97.06 | 92.27 | 55.49 | 54654711 | 26248204 |
| ZH-Q30-40-1 | 34439422 | 10.33 | 98.05 | 94.55 | 57.54 | 32300193 | 20031930 |
| ZH-Q30-40-2 | 50044485 | 15.01 | 96.88 | 91.92 | 56.17 | 44439237 | 17050996 |
| ZH-Q30-40-3 | 47191054 | 14.16 | 96.57 | 91.24 | 57.02 | 41279353 | 10609715 |
| ZH-Q30-40-4 | 36291095 | 10.89 | 97.91 | 94.1 | 54.05 | 33786874 | 19712266 |
| ZH-Q30-40-5 | 34707079 | 10.41 | 97.56 | 93.27 | 57.05 | 31941327 | 10628724 |
| ZH-Q40-50-1 | 39180448 | 11.75 | 97.92 | 94.15 | 54.82 | 36453033 | 22927945 |
| ZH-Q40-50-2 | 38261891 | 11.48 | 97.51 | 93.14 | 57.48 | 35182999 | 10282353 |
| ZH-Q40-50-3 | 36257235 | 10.88 | 97.46 | 92.9 | 54.13 | 33223736 | 11163701 |
| ZH-Q40-50-4 | 36519819 | 10.96 | 97.63 | 93.35 | 55.16 | 33685336 | 17517547 |
| ZH-Q40-50-5 | 34507721 | 10.35 | 96.96 | 91.81 | 54.81 | 30964271 | 13893635 |
| ZH-Q50-60-1 | 37674354 | 11.3 | 97.72 | 93.66 | 54.19 | 34762109 | 23723682 |
| ZH-Q50-60-2 | 44974552 | 13.49 | 97.88 | 94.12 | 56.5 | 41897125 | 21206649 |
| ZH-Q50-60-3 | 35312547 | 10.59 | 97.27 | 92.66 | 56.42 | 32088130 | 8410023 |
| ZH-Q50-60-4 | 37577731 | 11.27 | 97.43 | 92.95 | 54.19 | 34436972 | 11470757 |
| ZH-Q50-60-5 | 36890109 | 11.07 | 97.59 | 93.41 | 54.48 | 34037053 | 10034897 |
| ZH-Q60-80-1 | 37855160 | 11.36 | 97.42 | 93.16 | 53.98 | 34589347 | 12641880 |
| ZH-Q60-80-2 | 36482704 | 10.94 | 97.55 | 93.38 | 54.32 | 33581269 | 11988175 |
| ZH-Q60-80-3 | 34799733 | 10.44 | 97.6 | 93.49 | 56.38 | 32065315 | 12895421 |
| ZH-Q60-80-4 | 34404399 | 10.32 | 97.58 | 93.53 | 53.98 | 31668849 | 9574563 |
| ZH-Q60-80-5 | 37371641 | 11.21 | 97.21 | 92.65 | 54.65 | 33835609 | 12333269 |
| ZH-Q80-100-1 | 35313245 | 10.59 | 97.59 | 93.45 | 56.97 | 32568181 | 8940087 |
| ZH-Q80-100-2 | 35929383 | 10.78 | 97.64 | 93.52 | 53.34 | 33208473 | 10346104 |
| ZH-Q80-100-3 | 34936488 | 10.48 | 97.32 | 92.81 | 55.95 | 31773323 | 9216940 |
| ZH-Q80-100-4 | 37285450 | 11.19 | 97.54 | 93.34 | 55.22 | 34256599 | 10738277 |
| ZH-Q80-100-5 | 40907879 | 12.27 | 97.33 | 92.81 | 55.64 | 37304899 | 7392991 |

**Table S2** Multiple response permutation procedure (MRPP) analysis of functional profiles related to methane, nitrogen and sulphur cycling among different depths. Values for significant levels are denoted with *(0.01 < *P* < 0.05), **(0.001 < *P* < 0.01) and ***(*P* < 0.001).

| Depth | 0-5 | 5-10 | 10-15 | 15-20 | 20-30 | 30-40 | 40-50 | 50-60 | 60-80 | 80-100 |
| --- | --- | --- | --- | --- | --- | --- | --- | --- | --- | --- |
| 0-5 |  | 0.232 | 0.2 | 0.097 | * | ** | * | ** | ** | ** |
| 5-10 |  |  | 0.908 | 0.777 | 0.241 | ** | ** | ** | ** | * |
| 10-15 |  |  |  | 0.805 | 0.232 | * | ** | ** | * | * |
| 15-20 |  |  |  |  | 0.595 | * | * | * | * | * |
| 20-30 |  |  |  |  |  | 0.357 | 0.059 | 0.06 | * | * |
| 30-40 |  |  |  |  |  |  | 0.295 | 0.381 | 0.272 | 0.103 |
| 40-50 |  |  |  |  |  |  |  | 0.89 | 0.899 | 0.15 |
| 50-60 |  |  |  |  |  |  |  |  | 0.924 | 0.239 |
| 60-80 |  |  |  |  |  |  |  |  |  | 0.246 |
| 80-100 |  |  |  |  |  |  |  |  |  |  |

**Table S3** Analysis of similarity (ANOSIM) based on functional profiles related to methane, nitrogen and sulphur cycling among different depths. Values for significant levels are denoted with *(0.01 < *P* < 0.05), **(0.001 < *P* < 0.01) and ***(*P* < 0.001).

| Depth | 0-5 | 5-10 | 10-15 | 15-20 | 20-30 | 30-40 | 40-50 | 50-60 | 60-80 | 80-100 |
| --- | --- | --- | --- | --- | --- | --- | --- | --- | --- | --- |
| 0-5 |  | 0.173 | 0.153 | 0.059 | * | * | ** | ** | * | * |
| 5-10 |  |  | 0.787 | 0.92 | 0.26 | ** | ** | ** | ** | * |
| 10-15 |  |  |  | 0.976 | 0.325 | ** | ** | ** | ** | ** |
| 15-20 |  |  |  |  | 0.706 | 0.118 | * | * | * | * |
| 20-30 |  |  |  |  |  | 0.855 | 0.077 | 0.147 | 0.051 | * |
| 30-40 |  |  |  |  |  |  | 0.327 | 0.391 | 0.271 | 0.056 |
| 40-50 |  |  |  |  |  |  |  | 0.893 | 0.902 | 0.07 |
| 50-60 |  |  |  |  |  |  |  |  | 0.926 | 0.172 |
| 60-80 |  |  |  |  |  |  |  |  |  | 0.075 |
| 80-100 |  |  |  |  |  |  |  |  |  |  |

**Table S4** The vertical distribution of physicochemical characteristics of mangrove sediment samples (mean ± SD).

|  | Depth (cm) | PH | Temperature  (℃) | Salinity  （%） | SO_4_^2-^  (mg/l) | AVS  (mmol/l dry weight) | S_0_  (mg/kg) | NO_3_^-^  (μg N/L) | NO_2_^-^  (μg N/L) | NH_4_^+^  (mg N/L) | TC  （%） | TN  （%） | TS  （%） |
| --- | --- | --- | --- | --- | --- | --- | --- | --- | --- | --- | --- | --- | --- |
| KO1 | 0-5 | 6.39±0.12^c^ | 19.52±0.19^ab^ | 1.26±0.05^a^ | 596.17±75.03^a^ | 19.47±2.80^a^ | 448.01±183.12^a^ | 133.98±21.29^ab^ | 91.78±11.45^a^ | 3.30±0.39^b^ | 3.23±0.43^a^ | 0.28±0.03^ab^ | 0.94±0.12^a^ |
| KO2 | 5-10 | 6.52±0.13^bc^ | 19.22±0.11^b^ | 0.97±0.08^ab^ | 260.83±16.38^ab^ | 14.93±1.08^b^ | 717.05±257.82^a^ | 139.63±7.31^ab^ | 58.91±7.38^b^ | 4.74±0.87^a^ | 3.62±0.52^a^ | 0.31±0.04^a^ | 0.82±0.10^a^ |
| KO3 | 10-15 | 6.45±0.10^bc^ | 19.20±0.17^b^ | 1.01±0.16^ab^ | 155.33±9.95^ab^ | 16.99±3.00^ab^ | 1508.76±661.03^a^ | 88.29±21.34^b^ | 86.24±16.29^ab^ | 3.01±0.32^bc^ | 3.63±0.70^a^ | 0.31±0.05^a^ | 0.73±0.08^a^ |
| KO4 | 15-20 | 6.55±0.05^bc^ | 19.28±0.17^b^ | 0.98±0.14^ab^ | 88.17±4.29^b^ | 13.40±0.61^b^ | 2014.12±1088.38^a^ | 101.13±11.97^b^ | 47.12±16.30^b^ | 2.05±0.13^c^ | 3.41±0.64^a^ | 0.29±0.05^ab^ | 0.77±0.11^a^ |
| KO5 | 20-30 | 6.75±0.09^b^ | 19.48±0.19^b^ | 0.92±0.10^ab^ | 115.00±13.21^b^ | 16.06±1.30^ab^ | 1881.02±746.27^a^ | 229.97±34.37^a^ | 81.00±5.61^ab^ | 2.10±0.25^c^ | 3.05±0.55^ab^ | 0.26±0.04^ab^ | 0.84±0.15^a^ |
| KO6 | 30-40 | 6.88±0.15^ab^ | 19.72±0.15^ab^ | 0.91±0.12^ab^ | 95.50±9.90^b^ | 16.12±0.99^ab^ | 1168.65±642.71^a^ | 148.35±15.34^ab^ | 85.09±10.15^ab^ | 1.86±0.10^c^ | 2.69±0.48^ab^ | 0.23±0.04^ab^ | 0.91±0.18^a^ |
| KO7 | 40-50 | 7.07±0.07^ab^ | 19.76±0.17^ab^ | 0.87±0.09^b^ | 133.51±38.20^b^ | 0.80±0.14^c^ | 937.59±470.98^a^ | 213.14±12.11^a^ | 95.48±11.49^a^ | 1.84±0.57^c^ | 2.40±0.35^ab^ | 0.20±0.02^b^ | 1.02±0.25^a^ |
| KO8 | 50-60 | 7.11±0.11^a^ | 20.12±0.19^a^ | 0.84±0.13^b^ | 55.52±6.67^b^ | 0.84±0.17^c^ | 432.77±172.73^a^ | 227.92±5.27^a^ | 89.71±4.66^a^ | 2.38±0.39^bc^ | 2.45±0.35^ab^ | 0.20±0.03^b^ | 1.06±0.26^a^ |
| KO9 | 60-80 | 7.17±0.12^a^ | 20.12±0.31^a^ | 0.87±0.13^b^ | 64.00±1.92^b^ | 0.70±0.04^c^ | 270.91±66.21^a^ | 267.96±14.99^a^ | 86.86±5.44^ab^ | 2.56±0.30^bc^ | 2.39±0.25^ab^ | 0.19±0.03^b^ | 1.11±0.18^a^ |
| KO10 | 80-100 | 7.17±0.13^a^ | 20.12±0.39^a^ | 0.88±0.18^b^ | 30.48±1.91^b^ | 0.80±0.07^c^ | 312.73±38.42^a^ | 294.91±30.16^a^ | 103.95±6.25^a^ | 2.12±0.17^c^ | 1.75±0.12^b^ | 0.14±0.01^b^ | 1.03±0.16^a^ |

*Note:* Small letters mean a statistical significance (*P* < 0.05) among different depths. AVS: acid volatile sulfide; TC: total carbon; TN: total nitrogen; TS: total sulphur.

| Gene category | PH | Temperature  (℃) | Salinity  （%） | SO_4_^2-^  (mg/l) | AVS  (mmol/l) | S_0_  (mg/kg) | NO_3_^-^  (μg N/L) | NO_2_^-^  (μg N/L) | NH_4_^+^  (mg N/L) | TC  （%） | TN  （%） | TS  （%） |
| --- | --- | --- | --- | --- | --- | --- | --- | --- | --- | --- | --- | --- |
| Denitrification | 0.3564  (**0.001**) | 0.0112  (0.437) | 0.0009  (0.499) | 0.1057  (0.105) | 0.2698  (**0.001**) | 0.0500  (0.754) | 0.2393  (**0.001**) | 0.1610  (**0.016**) | 0.0301  (0.608) | 0.1028  (0.055) | 0.1060  (**0.025**) | 0.0507  (0.186) |
| Anammox | 0.2350  (**0.004**) | 0.0309  (0.314) | 0.1151  (**0.029**) | 0.0312  (0.295) | 0.1005  (**0.027**) | 0.0297  (0.313) | 0.0877  (0.098) | 0.0959  (0.14) | 0.0813  (0.849) | 0.0938  (0.094) | 0.1078  (0.057) | 0.0251  (0.613) |
| DNRA | 0.3835  (**0.001**) | 0.0068  (0.408) | 0.0324  (0.299) | 0.3290  (**0.009**) | 0.1877  (**0.002**) | 0.0829  (0.831) | 0.1668  (**0.034**) | 0.2045  (**0.016**) | 0.0260  (0.498) | 0.0592  (0.204) | 0.0588  (0.166) | 0.0248  (0.336) |
| N_2_ fixation | 0.1060  (**0.038**) | 0.0409  (0.259) | 0.0585  (0.126) | 0.2918  (**0.001**) | 0.2862  (**0.001**) | 0.0350  (0.639) | 0.1141  (**0.037**) | 0.0911  (0.079) | 0.0877  (0.113) | 0.1670  (**0.005**) | 0.1852  (**0.002**) | 0.0315  (0.692) |
| Dissimilatory sulphur reduction and oxidation | 0.1608  (**0.032**) | 0.0533  (0.681) | 0.0372  (0.697) | 0.1286  (0.111) | 0.0308  (0.216) | 0.0470  (0.269) | 0.0805  (0.164) | 0.2077  (**0.02**) | 0.0701  (0.781) | 0.1616  (0.055) | 0.1213  (**0.049**) | 0.0456  (0.253) |
| Sulphur reduction | 0.3861  (**0.001**) | 0.0162  (0.508) | 0.0160  (0.369) | 0.3008  (**0.015**) | 0.1906  (**0.002**) | 0.0986  (0.904) | 0.1575  (**0.033**) | 0.1613  (**0.036**) | 0.0050  (0.406) | 0.0107  (0.355) | 0.0151  (0.516) | 0.0742  (0.16) |
| Sox system | 0.3524  (**0.001**) | 0.1270  (**0.048**) | 0.0463  (0.185) | 0.1668  (**0.035**) | 0.3979  (**0.001**) | 0.1199  (0.986) | 0.3335  (**0.001**) | 0.1689(  **0.013**) | 0.0398  (0.685) | 0.0914  (0.063) | 0.1409  (**0.012**) | 0.0408  (0.216) |
| sulphur oxidation | 0.2962  (**0.001**) | 0.1276  (**0.014**) | 0.1129  (**0.01**) | 0.2296  (**0.001**) | 0.4458  (**0.001**) | 0.0574  (0.841) | 0.3024  (**0.001**) | 0.1276  (**0.011**) | 0.0071  (0.505) | 0.1992  (**0.001**) | 0.2815  (**0.001**) | 0.0430  (0.802) |
| Aerobic oxidation of methane | 0.4084  (**0.001**) | 0.0610  (0.196) | 0.0634  (0.132) | 0.3327  (**0.005**) | 0.3067  (**0.001**) | 0.1096  (0.954) | 0.2437  (**0.003**) | 0.1849  (**0.023**) | 0.0061  (0.434) | 0.0636  (0.163) | 0.0832  (0.097) | 0.0282  (0.31) |
| Methanogenesis | 0.4289  (**0.001**) | 0.0214  (0.337) | 0.0314  (0.264) | 0.2868  (**0.003**) | 0.2918  (**0.001**) | 0.0966  (0.918) | 0.2194  (**0.005**) | 0.1672  (**0.019**) | 0.0066  (0.46) | 0.0762  (0.133) | 0.0837  (0.092) | 0.0489  (0.197) |

**Table S5** Summary statistics for Mantel tests of correlations between metabolic pathways and environmental factors.

*Note:* Values are correlation coefficients with P values in brackets (*P* < 0.05 in bold).

**Table S6** Summary of correlations between functional groups and keystones.

| **Keystone** | **Interaction groups** | **Betweeness** | **Direction** |
| --- | --- | --- | --- |
| *Sulfuricaulis limicola (surface layer)* | *Bradyrhizobiaceae bacterium SG-6C* | 61.17460317 | 1 |
|  | *Bradyrhizobium taxid(374)* | 187.6666667 | 1 |
|  | *Bradyrhizobium oligotrophicum S58* | 64.12380952 | 1 |
|  | *Bradyrhizobium sp. ORS* | 76.91349206 | 1 |
|  | *Methyloceanibacter caenitepidi* | 74.43730159 | 1 |
|  | *Pseudolabrys taiwanensis* | 43.64285714 | 1 |
|  | *Rhizobiales taxid (356)* | 373.1190476 | 1 |
|  | *Rhodoplanes sp. Z2-YC6860* | 120.3333333 | 1 |
|  | *Stella humosa* | 105.8007937 | 1 |
|  | *unclassified Bradyrhizobium* | 56.60396825 | 1 |
| *Betaproteobacteria taxid(28216) (middle layer)* | *Acidiferrobacter sp. SPIII 3* | 422.7364137 | 1 |
|  | *Alphaproteobacteria taxid(28211)* | 200.8745329 | 1 |
|  | *Anaeromyxobacter dehalogenans 2CP-C* | 139.1634994 | 1 |
|  | *Anaeromyxobacter sp. Fw109-5* | 297.998284 | 1 |
|  | *Ancylobacter sp. TS-1* | 285.9247463 | 1 |
|  | *Aquabacterium olei* | 418.7064161 | 1 |
|  | *Azospirillum taxid 191* | 214.3621066 | 1 |
|  | *Betaproteobacteria bacterium GR16-43* | 39.56250684 | 1 |
|  | *Bradyrhizobium taxid(374)* | 163.8546449 | 1 |
|  | *Bradyrhizobium erythrophlei* | 81.62162263 | 1 |
|  | *Bradyrhizobium sp. 58S1* | 97.81294809 | 1 |
|  | *Bradyrhizobium sp. BTAi1* | 193.7818279 | 1 |
|  | *Bradyrhizobium zhanjiangense* | 145.6329102 | 1 |
|  | *Burkholderia taxid(32008)* | 195.9464094 | 1 |
|  | *Burkholderia cepacia complex* | 124.1616815 | 1 |
|  | *Burkholderia sp. DHOD12* | 175.5199592 | 1 |
|  | *Burkholderia stagnalis* | 160.3713164 | 1 |
|  | *Burkholderiaceae taxid(119060)* | 165.4540364 | 1 |
|  | *Burkholderiales bacterium GJ-E10* | 1230.069034 | 1 |
|  | *Burkholderiales bacterium JOSHI 001* | 102.1123937 | 1 |
|  | *Candidatus Filomicrobium marinum* | 59.72677178 | 1 |
|  | *Candidatus Promineofilum breve* | 165.3807778 | 1 |
|  | *Candidatus Symbiobacter mobilis* | 255.0044661 | 1 |
|  | *Celeribacter indicus* | 1567.64887 | 1 |
|  | *Cupriavidus gilardii CR3* | 90.34454298 | 1 |
|  | *Cupriavidus metallidurans* | 266.4934203 | 1 |
|  | *Cupriavidus taiwanensis* | 127.8620869 | 1 |
|  | *Desulfitobacterium hafniense* | 448.7392246 | 1 |
|  | *Desulfocurvibacter africanus subsp.* | 510.1115552 | -1 |
|  | *Gammaproteobacteria taxid(1236)* | 556.9880575 | 1 |
|  | *Halorhodospira halochloris* | 120.2375618 | 1 |
|  | *Hyphomicrobium nitrativorans NL23* | 235.7760125 | 1 |
|  | *Methyloceanibacter sp. wino2* | 220.3156467 | 1 |
|  | *Methylocystis bryophila* | 494.9515661 | 1 |
|  | *Microvirgula aerodenitrificans* | 543.7331234 | 1 |
|  | *Nitrospira japonica* | 43.87087914 | 1 |
|  | *Nitrospira moscoviensis* | 78.15803573 | 1 |
|  | *Pandoraea thiooxydans* | 87.47978955 | 1 |
|  | *Paraburkholderia rhizoxinica HKI* | 58.72743396 | 1 |
|  | *Phreatobacter sp. NMCR1094* | 122.7915884 | 1 |
|  | *Pseudolabrys sp. FHR47* | 99.32104844 | 1 |
|  | *Pseudolabrys taiwanensis* | 129.2683113 | 1 |
|  | *Pseudomonas protegens* | 43.87087914 | 1 |
|  | *Pseudorhodoplanes sinuspersici* | 133.1747092 | 1 |
|  | *Ralstonia insidiosa* | 313.2213185 | 1 |
|  | *Ralstonia solanacearum* | 195.4115077 | 1 |
|  | *Rhizobiales taxid(356)* | 124.3549573 | 1 |
|  | *Rhodopseudomonas palustris BisA53* | 829.8027115 | 1 |
|  | *Rhodospirillaceae bacterium R5913* | 148.209782 | 1 |
|  | *Sorangium cellulosum* | 80.84117071 | 1 |
|  | *Sphaerotilus natans subsp.* | 238.703802 | 1 |
|  | *Sphingomonas taxid(13687)* | 306.1454149 | 1 |
|  | *Sporosarcina sp. P37* | 120.2375618 | 1 |
|  | *Sterolibacteriaceae bacterium M52* | 330.9111567 | 1 |
|  | *Sulfuricella denitrificans skB26* | 281.3504831 | 1 |
|  | *Sulfuritalea hydrogenivorans sk43H* | 114.2097713 | 1 |
|  | *Thauera aromatica K172* | 523.6797143 | 1 |
|  | *Thauera chlorobenzoica* | 192.9595801 | 1 |
|  | *Thauera sp. MZ1T* | 160.5445065 | 1 |
|  | *Thermaerobacter sp. FW80* | 223.0478106 | 1 |
|  | *unclassified Bradyrhizobium* | 158.2715567 | 1 |
|  | *unclassified Streptomyces* | 73.45873278 | 1 |
| *Desulfomicrobium orale DSM (middle layer)* | *Achromobacter spanius* | 435.5796406 | -1 |
|  | *Arenimonas daejeonensis* | 480.8639736 | -1 |
|  | *Bordetella taxid(517)* | 744.3177047 | -1 |
|  | *Burkholderia cenocepacia* | 275.8269673 | -1 |
|  | *Chromatiaceae taxid(1046)* | 325.1680208 | -1 |
|  | *Hydrogenophaga taxid(47420)* | 108.776537 | -1 |
|  | *Melaminivora sp. SC2-9* | 1164.875136 | -1 |
|  | *Methyloceanibacter sp. wino2* | 1427.928704 | -1 |
|  | *Nocardioides sp. JS614* | 251.7813425 | -1 |
|  | *Polaromonas sp. JS666* | 256.9074574 | -1 |
|  | *Sphingomonadaceae taxid(41297)* | 328.2065308 | -1 |
|  | *Sulfuriflexus mobilis* | 434.1440792 | -1 |
|  | *Sulfuritortus calidifontis* | 231.2605795 | -1 |
|  | *Thiolapillus brandeum* | 916.3502986 | -1 |
| *Desulfovibrio desulfuricans ND132 (middle layer)* | *Allochromatium vinosum DSM* | 229.0540638 | 1 |
|  | *Burkholderia ubonensis* | 334.7756344 | 1 |
|  | *Deltaproteobacteria taxid(28221)* | 245.965672 | 1 |
|  | *Desulfatibacillum aliphaticivorans* | 140.6824562 | 1 |
|  | *Desulfobacterium autotrophicum HRM2* | 111.3937388 | 1 |
|  | *Desulfobacula toluolica Tol2* | 202.0304908 | 1 |
|  | *Desulfococcus oleovorans Hxd3* | 93.85074602 | 1 |
|  | *Desulfohalobium retbaense DSM* | 585.3293245 | -1 |
|  | *Desulfovibrio fairfieldensis* | 80.97489537 | 1 |
|  | *Desulfovibrio sp. IOR2* | 567.3667987 | 1 |
|  | *Methanoculleus taxid(45989)* | 352.1132944 | 1 |
|  | *Methanoculleus marisnigri JR1* | 142.9190526 | 1 |
|  | *Methanofollis liminatans DSM* | 334.962723 | 1 |
|  | *Methanolinea tarda NOBI-1* | 370.6460147 | 1 |
|  | *Pseudomonas xinjiangensis* | 130.2486172 | -1 |
|  | *Syntrophus aciditrophicus SB* | 674.9471227 | 1 |
|  | *Thioploca ingrica* | 214.0930059 | 1 |
| *Methanoregula boonei 6A8 (middle layer)* | *Achromobacter taxid(222)* | 264.2457692 | -1 |
|  | *Acidihalobacter prosperus* | 449.3580996 | 1 |
|  | *Azoarcus sp. SY39* | 490.7722312 | -1 |
|  | *Betaproteobacteria bacterium GR16-43* | 330.3772344 | -1 |
|  | *Bradyrhizobium sp. 58S1* | 224.8347062 | -1 |
|  | *Burkholderia sp. KK1* | 498.6190081 | -1 |
|  | *Celeribacter indicus* | 200.7583864 | -1 |
|  | *Chromatiaceae bacterium 2141T.STBD.0c.01a* | 741.3601046 | 1 |
|  | *Desulfovibrio gigas DSM* | 285.7345448 | 1 |
|  | *Halorhodospira halochloris* | 560.1029575 | -1 |
|  | *Methanoregula formicica SMSP* | 153.4622732 | 1 |
|  | *Methanosphaerula palustris E1-9c* | 111.209705 | 1 |
|  | *Methylovirgula ligni* | 496.6118271 | -1 |
|  | *Microbacteriaceae taxid(85023)* | 567.7311042 | -1 |
|  | *Microvirga sp. 17* | 760.2364939 | -1 |
|  | *Microvirgula aerodenitrificans* | 188.1968501 | -1 |
|  | *Nitrospira japonica* | 429.4262046 | -1 |
|  | *Pseudomonas protegens* | 429.4262046 | -1 |
|  | *Sporosarcina sp. P37* | 560.1029575 | -1 |
|  | *Sulfuricella denitrificans skB26* | 144.0290406 | -1 |
|  | *Sulfurivermis fontis* | 506.0466858 | 1 |
|  | *Thauera aromatica K172* | 263.2210609 | -1 |
|  | *unclassified Bradyrhizobium* | 208.3066835 | -1 |
| *Methylobacterium sp. 17SD2-17 (middle layer)* | *Acidihalobacter ferrooxidans* | 334.3136944 | -1 |
|  | *Alcaligenes faecalis* | 1014.863685 | -1 |
|  | *Azoarcus sp. BH72* | 778.1072766 | -1 |
|  | *Immundisolibacter cernigliae* | 1040.701032 | -1 |
|  | *Pandoraea apista* | 708.9745848 | -1 |
|  | *Paraburkholderia phymatum STM815* | 644.9852334 | -1 |
|  | *Sedimenticola thiotaurini* | 879.602917 | -1 |
|  | *gamma proteobacterium SS-5* | 1464 | -1 |
| *Proteobacteria taxid(1224) (middle layer)* | *Achromobacter xylosoxidans* | 107.7868361 | 1 |
|  | *Azoarcus sp. DN11* | 117.7365656 | 1 |
|  | *Azospirillum brasilense* | 87.14514066 | 1 |
|  | *Bacteria taxid(2)* | 53.35484408 | 1 |
|  | *Bradyrhizobium diazoefficiens* | 329.6318569 | 1 |
|  | *Bradyrhizobium guangxiense* | 59.58760211 | 1 |
|  | *Bradyrhizobium lablabi* | 239.7922301 | 1 |
|  | *Bradyrhizobium sp. BTAi1* | 147.0725035 | 1 |
|  | *Bradyrhizobium sp. CCGE-LA001* | 139.4099036 | 1 |
|  | *Bradyrhizobium zhanjiangense* | 112.0538977 | 1 |
|  | *Burkholderia lata* | 115.0772825 | 1 |
|  | *Candidatus Promineofilum breve* | 107.95466 | 1 |
|  | *Candidatus Solibacter usitatus* | 635.8007959 | 1 |
|  | *Cupriavidus metallidurans* | 82.27991306 | 1 |
|  | *Desulfitobacterium hafniense* | 59.27978981 | 1 |
|  | *Gammaproteobacteria taxid(1236)* | 197.352874 | 1 |
|  | *Gemmatirosa kalamazoonesis* | 134.1842292 | 1 |
|  | *Methylobacterium sp. 17Sr1-43* | 99.1621325 | 1 |
|  | *Oscillibacter valericigenes Sjm18-20* | 724.0780643 | 1 |
|  | *Pandoraea thiooxydans* | 190.8007277 | 1 |
|  | *Pseudolabrys sp. FHR47* | 218.2662621 | 1 |
|  | *Pseudolabrys taiwanensis* | 195.970922 | 1 |
|  | *Rhodococcus taxid 1827* | 359.0632686 | 1 |
|  | *Rhodoferax ferrireducens T118* | 143.8225628 | 1 |
|  | *Rhodoferax sp. CHu59-6-5* | 569.0168732 | 1 |
|  | *Rhodospirillum centenum SW* | 264.4136509 | 1 |
|  | *Sphingomonas taxid 13687* | 187.0969556 | 1 |
|  | *Stella humosa* | 87.36480888 | 1 |
|  | *Sterolibacteriaceae bacterium M52* | 93.97549197 | 1 |
|  | *Streptomyces taxid 1883* | 80.93861957 | 1 |
|  | *Sulfuricaulis limicola* | 192.0082358 | 1 |
|  | *Terrabacteria group* | 276.7242989 | 1 |
|  | *Thioalkalivibrio sulfidiphilus HL-EbGr7* | 57.32435895 | 1 |
|  | *unclassified Planctomycetes* | 263.6746522 | 1 |
| *Rhizobiales taxid(356) (middle layer)* | *Achromobacter spanius* | 138.4753869 | 1 |
|  | *Alphaproteobacteria taxid(28211)* | 100.5589031 | 1 |
|  | *Ancylobacter sp. TS-1* | 180.6918024 | 1 |
|  | *Azoarcus sp. KH32C* | 247.9727431 | 1 |
|  | *Betaproteobacteria taxid(28216)* | 124.3549573 | 1 |
|  | *Betaproteobacteria bacterium GR16-43* | 68.34931897 | 1 |
|  | *Bradyrhizobiaceae taxid(41294)* | 64.02636642 | 1 |
|  | *Bradyrhizobium taxid(374)* | 50.15488364 | 1 |
|  | *Bradyrhizobium erythrophlei* | 17.98795312 | 1 |
|  | *Bradyrhizobium sp. 2* | 223.3069609 | 1 |
|  | *Bradyrhizobium sp. BTAi1* | 131.4002052 | 1 |
|  | *Bradyrhizobium sp. SK17* | 88.75319468 | 1 |
|  | *Bradyrhizobium zhanjiangense* | 91.93443599 | 1 |
|  | *Burkholderia taxid(32008)* | 28.57996313 | 1 |
|  | *Burkholderia cenocepacia* | 258.8616641 | 1 |
|  | *Burkholderia cepacia* | 571.2059916 | 1 |
|  | *Burkholderia cepacia complex* | 11.26339631 | 1 |
|  | *Burkholderia lata* | 148.2279069 | 1 |
|  | *Burkholderia stagnalis* | 32.94294599 | 1 |
|  | *Burkholderia territorii* | 158.4427747 | 1 |
|  | *Burkholderiaceae taxid(119060)* | 46.96688135 | 1 |
|  | *Burkholderiales taxid(80840)* | 115.8569676 | 1 |
|  | *Burkholderiales bacterium JOSHI 001* | 7.7981012 | 1 |
|  | *Candidatus Filomicrobium marinum* | 23.03209181 | 1 |
|  | *Candidatus Promineofilum breve* | 110.9345429 | 1 |
|  | *Candidatus Thiodictyon syntrophicum* | 115.0068192 | 1 |
|  | *Cupriavidus gilardii CR3* | 49.96095846 | 1 |
|  | *Cupriavidus metallidurans* | 170.1763579 | 1 |
|  | *Cupriavidus necator N-1* | 66.80044928 | 1 |
|  | *Cupriavidus taiwanensis* | 40.84020134 | 1 |
|  | *Gammaproteobacteria taxid(1236)* | 291.2233161 | 1 |
|  | *Halorhodospira halochloris* | 99.01597799 | 1 |
|  | *Hyphomicrobium nitrativorans NL23* | 81.74656554 | 1 |
|  | *Methylibium petroleiphilum PM1* | 38.65589775 | 1 |
|  | *Methyloceanibacter sp. wino2* | 41.19261012 | 1 |
|  | *Nitrospira japonica* | 60.26403139 | 1 |
|  | *Nitrospira moscoviensis* | 42.93533952 | 1 |
|  | *Pandoraea thiooxydans* | 43.94889428 | 1 |
|  | *Paraburkholderia rhizoxinica HKI* | 55.98414753 | 1 |
|  | *Polaromonas sp. JS666* | 526.2986318 | 1 |
|  | *Pseudolabrys sp. FHR47* | 56.74934369 | 1 |
|  | *Pseudolabrys taiwanensis* | 63.90111 | 1 |
|  | *Pseudomonas taxid(286)* | 65.24250553 | 1 |
|  | *Pseudomonas protegens* | 60.26403139 | 1 |
|  | *Pseudorhodoplanes sinuspersici* | 118.8796335 | 1 |
|  | *Ralstonia taxid(48736)* | 121.1157412 | 1 |
|  | *Ralstonia insidiosa* | 259.7629751 | 1 |
|  | *Ralstonia solanacearum* | 188.3433703 | 1 |
|  | *Rhizobium leguminosarum* | 67.58219154 | 1 |
|  | *Rhodopseudomonas palustris* | 81.92452299 | 1 |
|  | *Rhodopseudomonas palustris BisA53* | 451.3698287 | 1 |
|  | *Rhodospirillaceae bacterium R5913* | 186.6679785 | 1 |
|  | *Sorangium cellulosum* | 56.33385863 | 1 |
|  | *Sphingomonadaceae taxid(41297)* | 184.3961154 | 1 |
|  | *Sporosarcina sp. P37* | 99.01597799 | 1 |
|  | *Sulfurifustis variabilis* | 156.4236983 | 1 |
|  | *Sulfuritalea hydrogenivorans sk43H* | 44.37880794 | 1 |
|  | *Thauera chlorobenzoica* | 101.4133141 | 1 |
|  | *Thermaerobacter sp. FW80* | 124.3459618 | 1 |
|  | *Variovorax paradoxus B4* | 112.1406862 | 1 |
|  | *Variovorax sp. HW608* | 176.6657306 | 1 |
|  | *unclassified Streptomyces* | 47.94736992 | 1 |
| *Thioalkalivibrio sulfidiphilus HL-EbGr7 (middle layer)* | *Achromobacter xylosoxidans* | 71.12759472 | 1 |
|  | *Azoarcus sp. DN11* | 71.12124861 | 1 |
|  | *Bacteria taxid 2* | 103.8636304 | 1 |
|  | *Bradyrhizobium diazoefficiens* | 150.7307191 | 1 |
|  | *Bradyrhizobium sp. CCGE-LA001* | 108.3644383 | 1 |
|  | *Candidatus Promineofilum breve* | 96.2043364 | 1 |
|  | *Candidatus Solibacter usitatus* | 241.2652293 | 1 |
|  | *Desulfitobacterium hafniense* | 48.037375 | 1 |
|  | *Gammaproteobacteria taxid(1236)* | 206.5086517 | 1 |
|  | *Gemmatirosa kalamazoonesis* | 65.72274164 | 1 |
|  | *Geobacter taxid(28231)* | 327.2855212 | 1 |
|  | *Methylovirgula ligni* | 245.687625 | 1 |
|  | *Proteobacteria taxid(1224)* | 57.32435895 | 1 |
|  | *Rhodospirillum centenum SW* | 114.1651471 | 1 |
|  | *Sterolibacteriaceae bacterium M52* | 131.8853352 | 1 |
|  | *Terrabacteria group* | 85.35203506 | 1 |

*Note:* 1 refers to positive correlation and -1 refers to negative correlation.

**Table S7** Summary of correlations between functional groups in the deep sediment.

| **Source** | **Target** | **Betweenness** | **Direction** |
| --- | --- | --- | --- |
| *Bacillus clausii* | *Bradyrhizobium taxid 374* | 2 | -1 |
| *Burkholderia sp. CCGE1003* | *Pelobacter propionicus DSM* | 2 | -1 |
| *Caldilinea aerophila DSM* | *unclassified Pseudomonas* | 4 | -1 |
| *Chryseobacterium bernardetii* | *Marichromatium purpuratum 984* | 8 | -1 |
| *Chryseobacterium bernardetii* | *Methyloceanibacter caenitepidi* | 6 | -1 |
| *Cupriavidus pauculus* | *Ruegeria pomeroyi DSS-3* | 2 | -1 |
| *Fictibacillus phosphorivorans* | *unclassified Pseudomonas* | 4 | -1 |
| *Gammaproteobacteria taxid 1236* | *Thioflavicoccus mobilis 8321* | 6 | 1 |
| *Hydrogenophaga sp. PBC* | *Sulfuritalea hydrogenivorans sk43H* | 2 | 1 |
| *Lautropia mirabilis* | *Methanoculleus taxid 45989* | 14 | -1 |
| *Lautropia mirabilis* | *Methanoregula boonei 6A8* | 24 | -1 |
| *Litoricola lipolytica* | *Methyloceanibacter sp. wino2* | 2 | -1 |
| *Marichromatium purpuratum 984* | *Microvirga sp. 17* | 6 | -1 |
| *Methanoculleus marisnigri JR1* | *Methanofollis liminatans DSM* | 6 | 1 |
| *Methanoculleus marisnigri JR1* | *Methanolinea tarda NOBI-1* | 8 | 1 |
| *Methanofollis liminatans DSM* | *Methanolinea tarda NOBI-1* | 3 | 1 |
| *Methanofollis liminatans DSM* | *Methanoregula boonei 6A8* | 13 | 1 |
| *Methanolinea tarda NOBI-1* | *Methanoregula boonei 6A8* | 12 | 1 |
| *Methanolinea tarda NOBI-1* | *Methanosarcina mazei* | 5 | -1 |
| *Methanoregula boonei 6A8* | *Methanoregula formicica SMSP* | 14 | 1 |
| *Methanoregula boonei 6A8* | *Methanosarcina mazei* | 9 | -1 |
| *Methanothrix soehngenii GP6* | *Planctomycetes bacterium ETA A1* | 2 | -1 |
| *Prosthecochloris sp. CIB* | *Thioflavicoccus mobilis 8321* | 6 | -1 |
| *Rhizobiales taxid 356* | *Thioflavicoccus mobilis 8321* | 6 | 1 |

1. **Supplementary Figures**


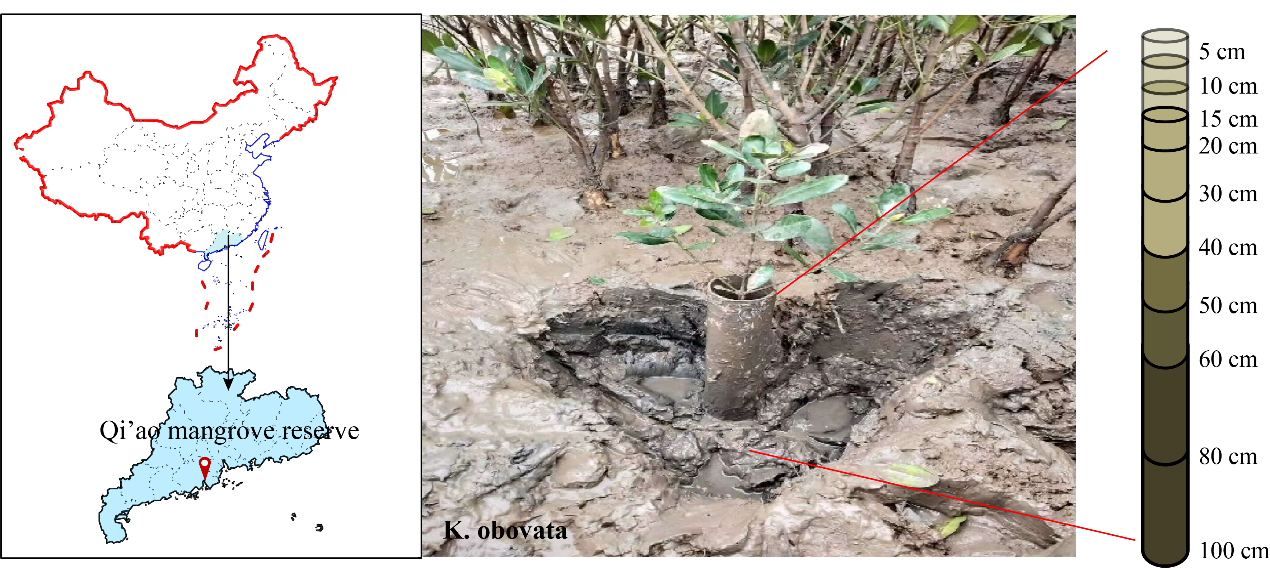


**Fig. S1** Sampling location at the Qi’ao Mangrove Reserve, Zhuhai, China, which is dominated by *Kandelia obovata*. In this study site, five sediment cores were collected using a custom-made sampler with a 100-cm depth and a 11-cm diameter. The sediment core was divided into 10-depths: 0-5, 5-10, 10-15, 15-20, 20-30, 30-40, 40-50, 50-60, 60-80, and 80-100 cm.


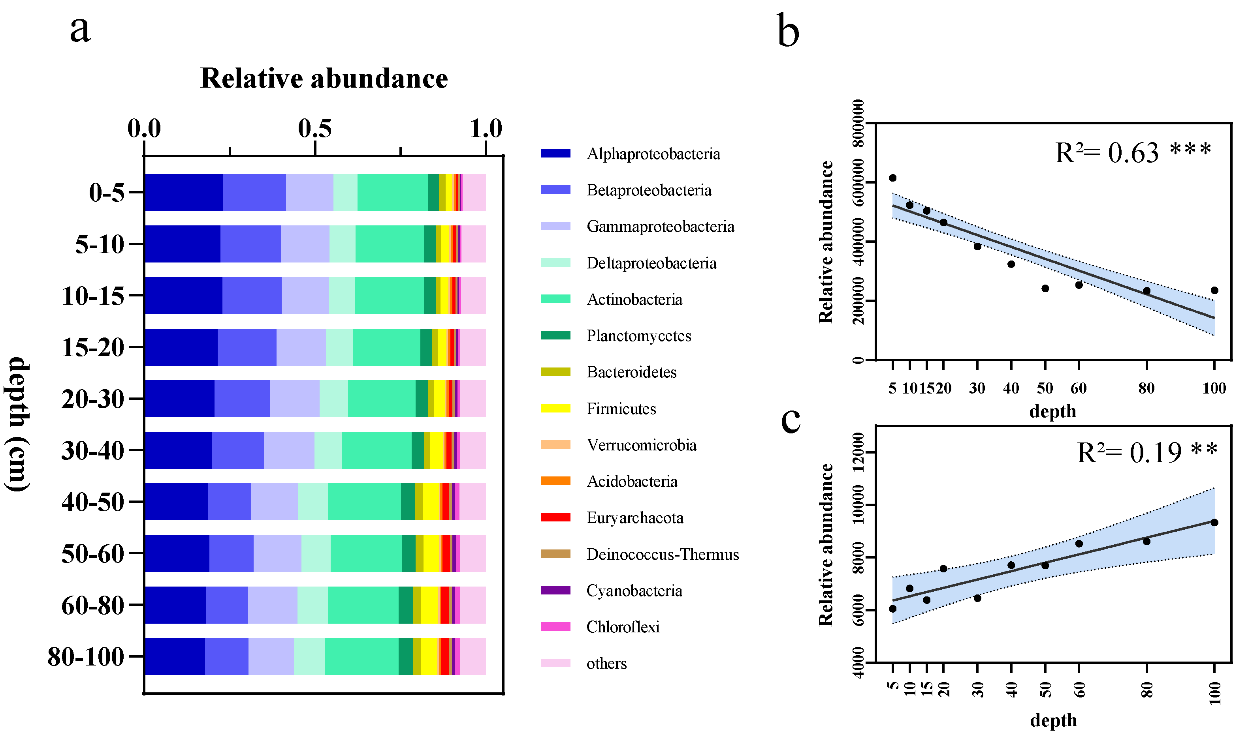


**Fig. S2** The depth-dependent profile of community structure in mangrove sediments. a. Stacked plot showing the depth-dependent composition of community using the phylogenetic annotation of metagenome sequencing reads. b. Relationships between the relative abundance of *Proteobacteria* and depth of mangrove sediments. c. Relationships between the relative abundance of *Euryarchaeota* and depth of mangrove sediments. Black lines and blue shaded areas represent linear regressions and 95% confidence intervals, respectively. R^2^ was obtained by linear regression analysis and significance levels are denoted with **(0.001 < *P* < 0.01) and ***(*P* < 0.001).


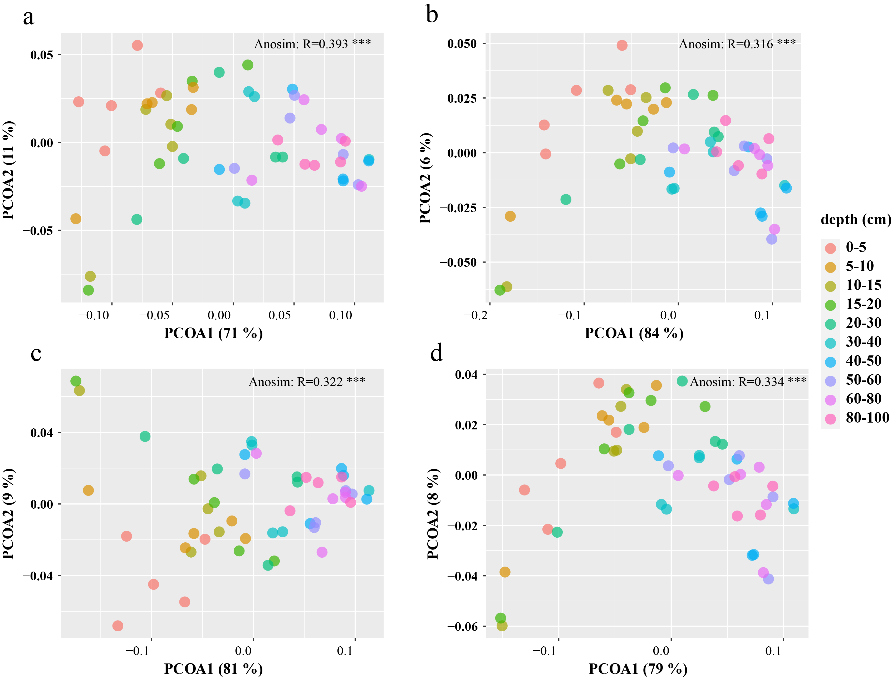


**Fig. S3** Principal coordinate analysis (PCoA) plot of all microbial functions (a), methane cycling (b), nitrogen cycling (c), and sulphur cycling (d). Underlying data are based on the abundance-weighted Bray–Curtis distance matrix derived from gene families and KO terms. The 50 samples shown in the 2D plane of each plot are spanned by their first two principal components. The similarity value and significance among the samples from different depths were examined via ANOSIM tests, which are shown in each plot.


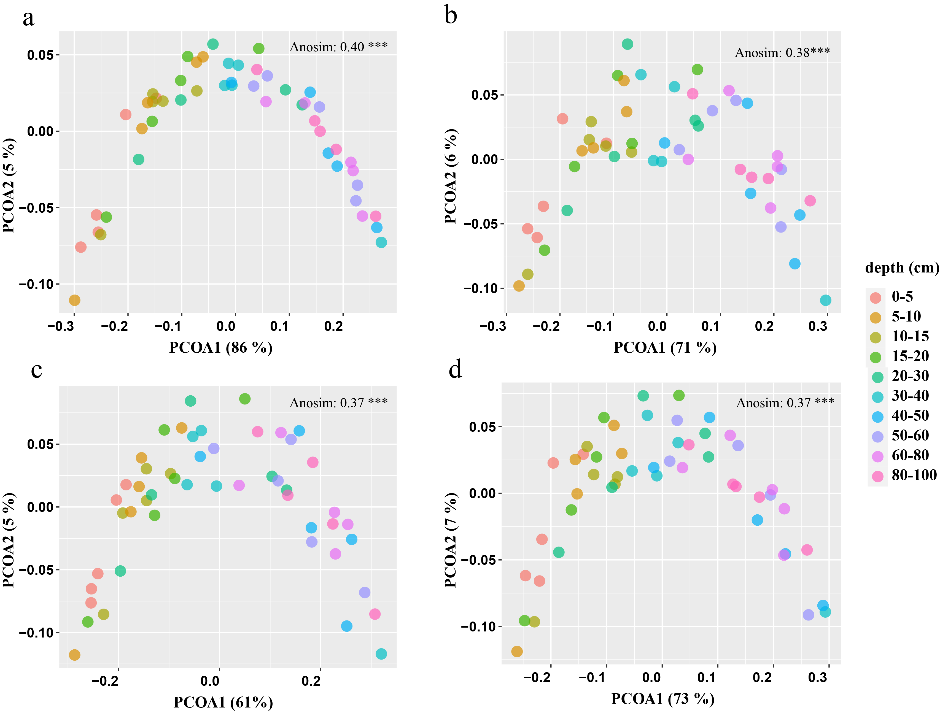


**Fig. S4** Principal coordinate analysis (PCoA) plot of all microbial communities (a), microbial communities of methane cycling (b), microbial communities of nitrogen cycling (c), and microbial communities of sulphur cycling (d). Underlying data are based on the abundance-weighted Bray–Curtis distance matrix derived from taxonomical profiles. The 50 samples shown in the 2D plane of each plot are spanned by their first two principal components. The similarity value and significance among the samples from different depth were examined via ANOSIM tests, which are shown in each plot.


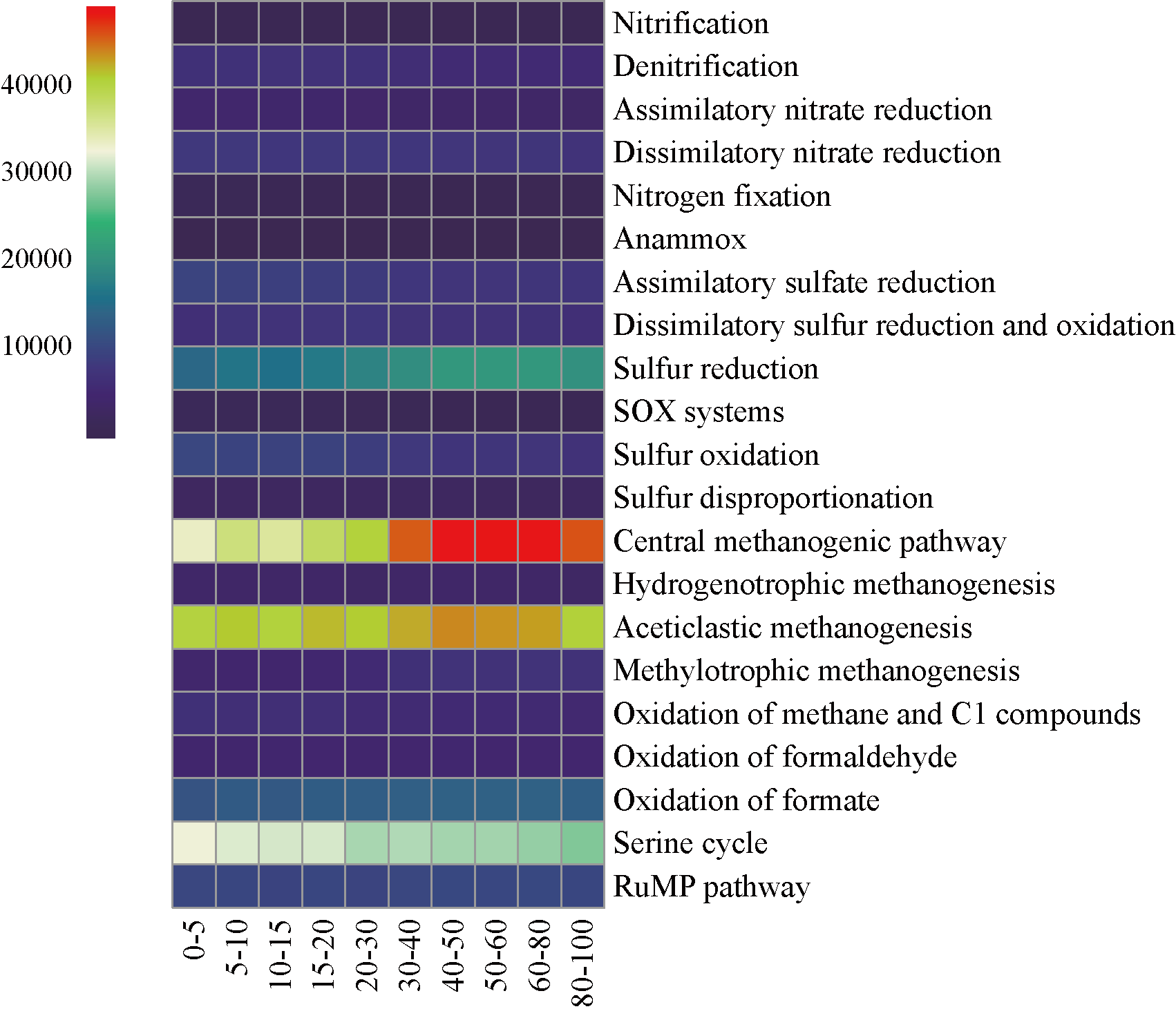


**Fig. S5** A heatmap plot of functional pathways for predicted open reading frames (ORFs) from metagenome sequencing reads for different depths of sediment samples.


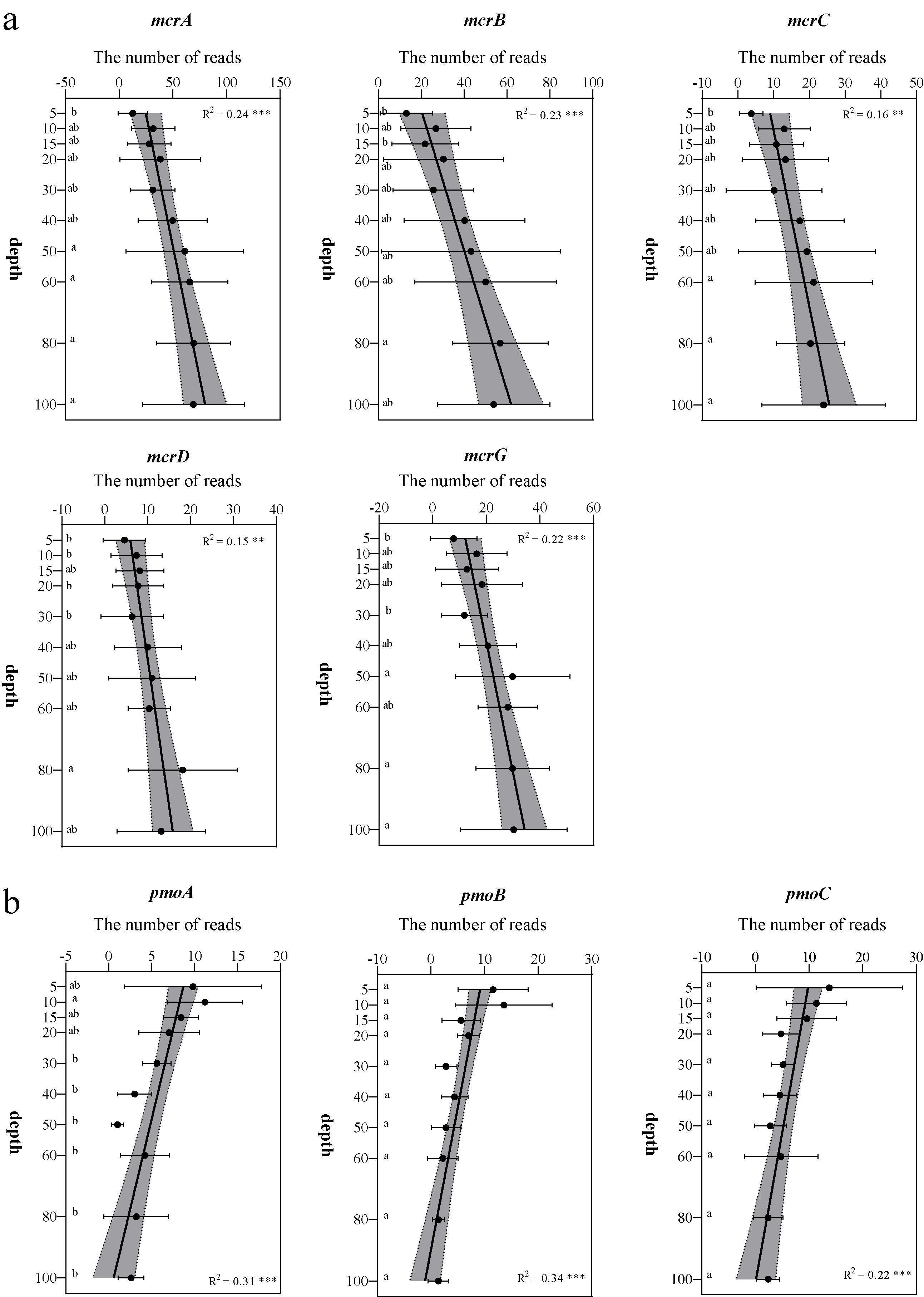


**Fig. S6** The vertical distribution of relative abundances of key gene families involved in methane cycling. Black lines and gray shaded areas represent linear regressions and 95% confidence intervals, respectively. R^2^ was obtained by linear regression analysis and significance levels are denoted with *(0.01 < *P* < 0.05), **(0.001 < *P* < 0.01) and ***(*P* < 0.001). Small letters mean a statistical significance (*P* < 0.05) among different depths.


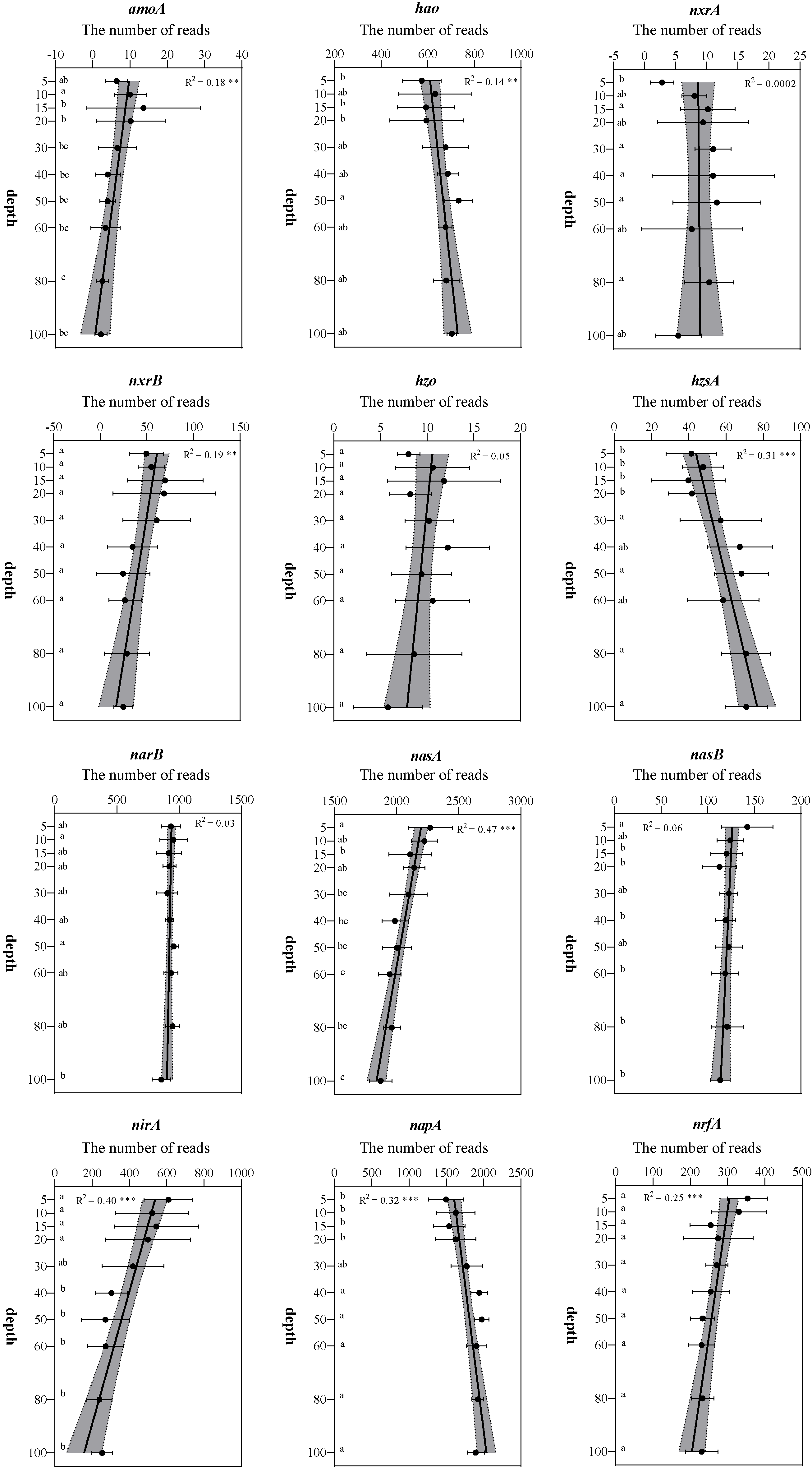

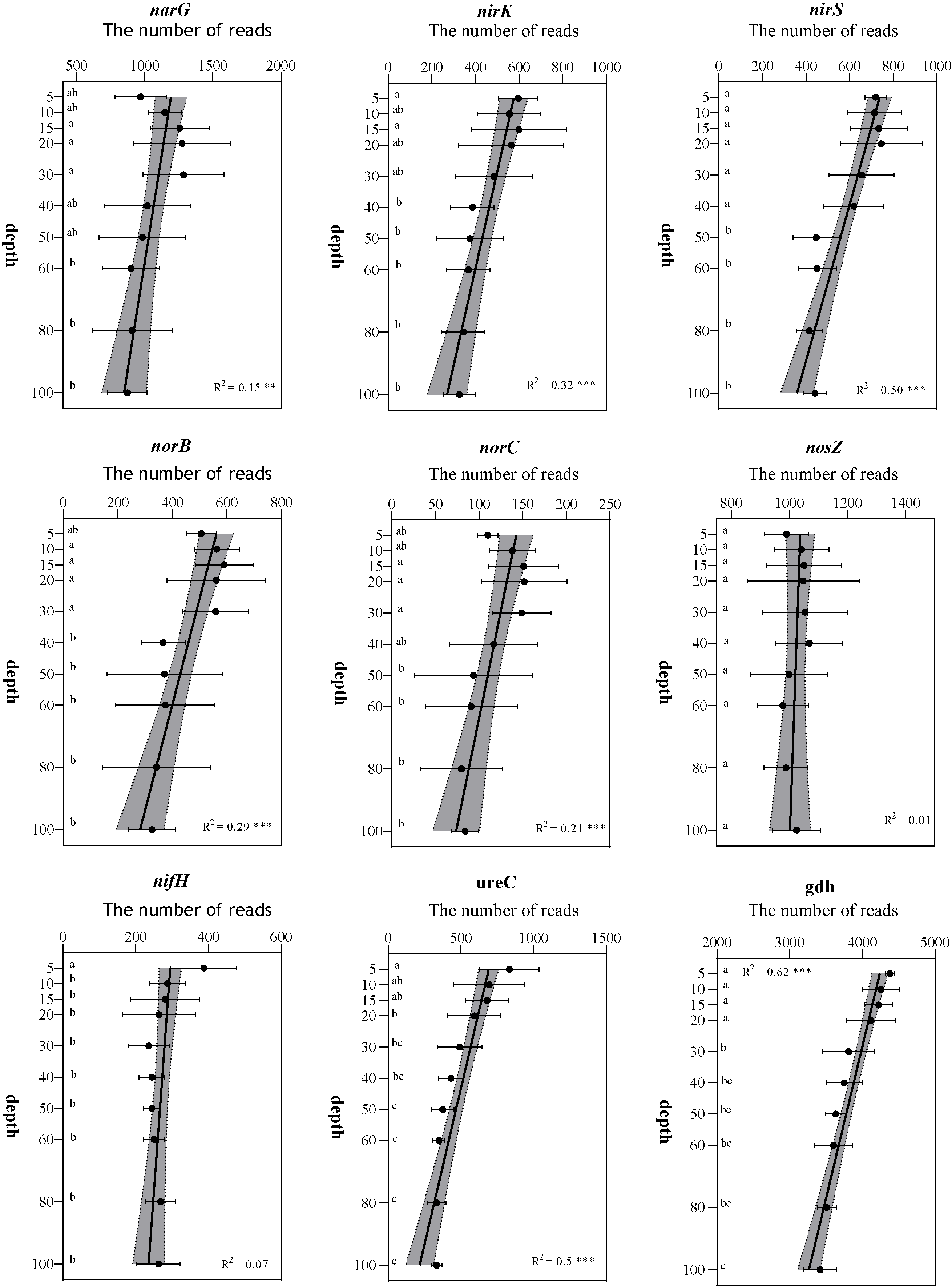


**Fig. S7** The vertical distribution of relative abundances of key gene families involved in nitrogen cycling. Black lines and gray shaded areas represent linear regressions and 95% confidence intervals, respectively. R^2^ was obtained by linear regression analysis and significance levels are denoted with *(0.01 < *P* < 0.05), **(0.001 < *P* < 0.01) and ***(*P* < 0.001). Small letters mean a statistical significance (*P* < 0.05) among different depths.


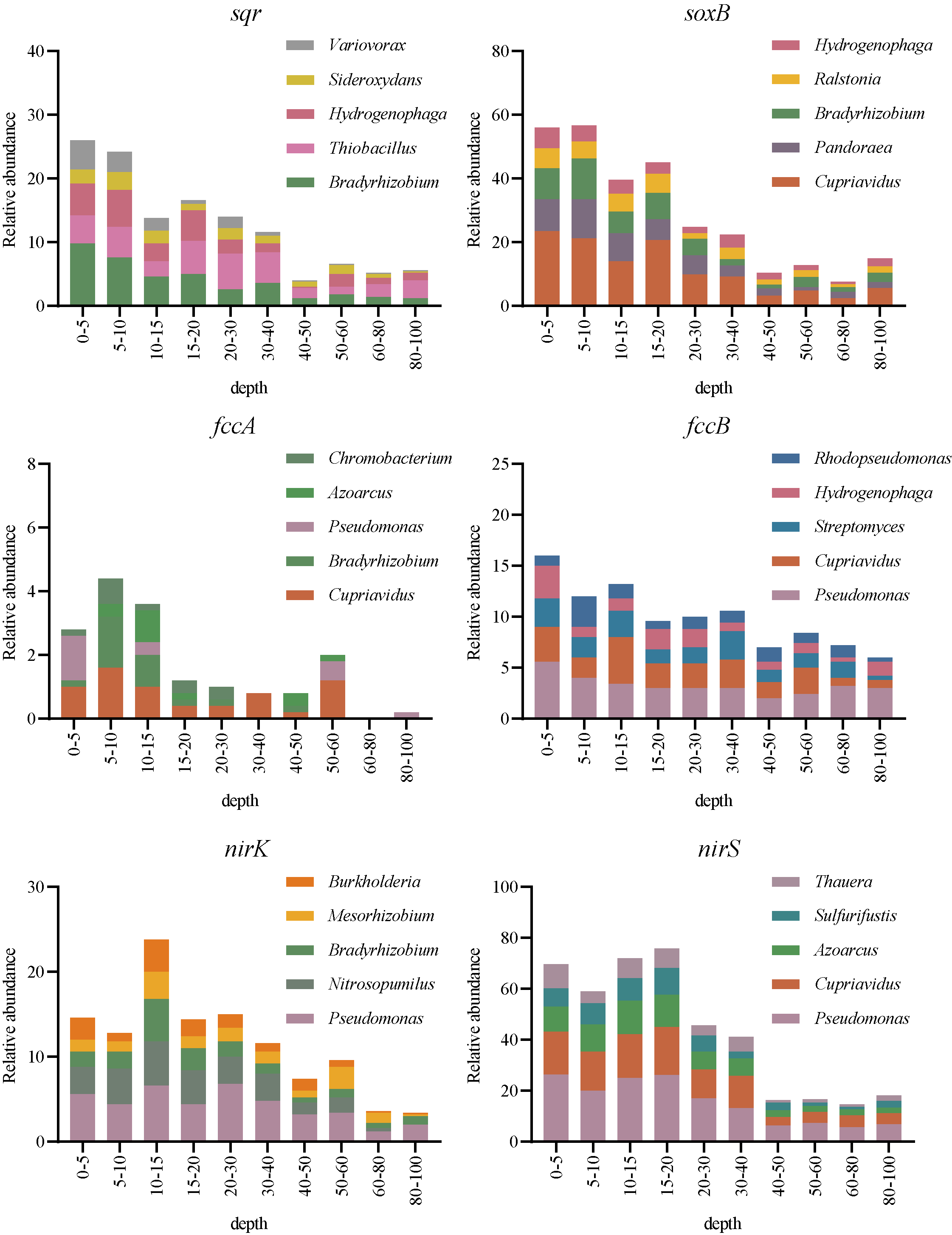


**Fig. S8** The vertical distribution of the relative abundance of key microbial taxa (top 5) responsible for genes involved in S oxidation and denitrification.


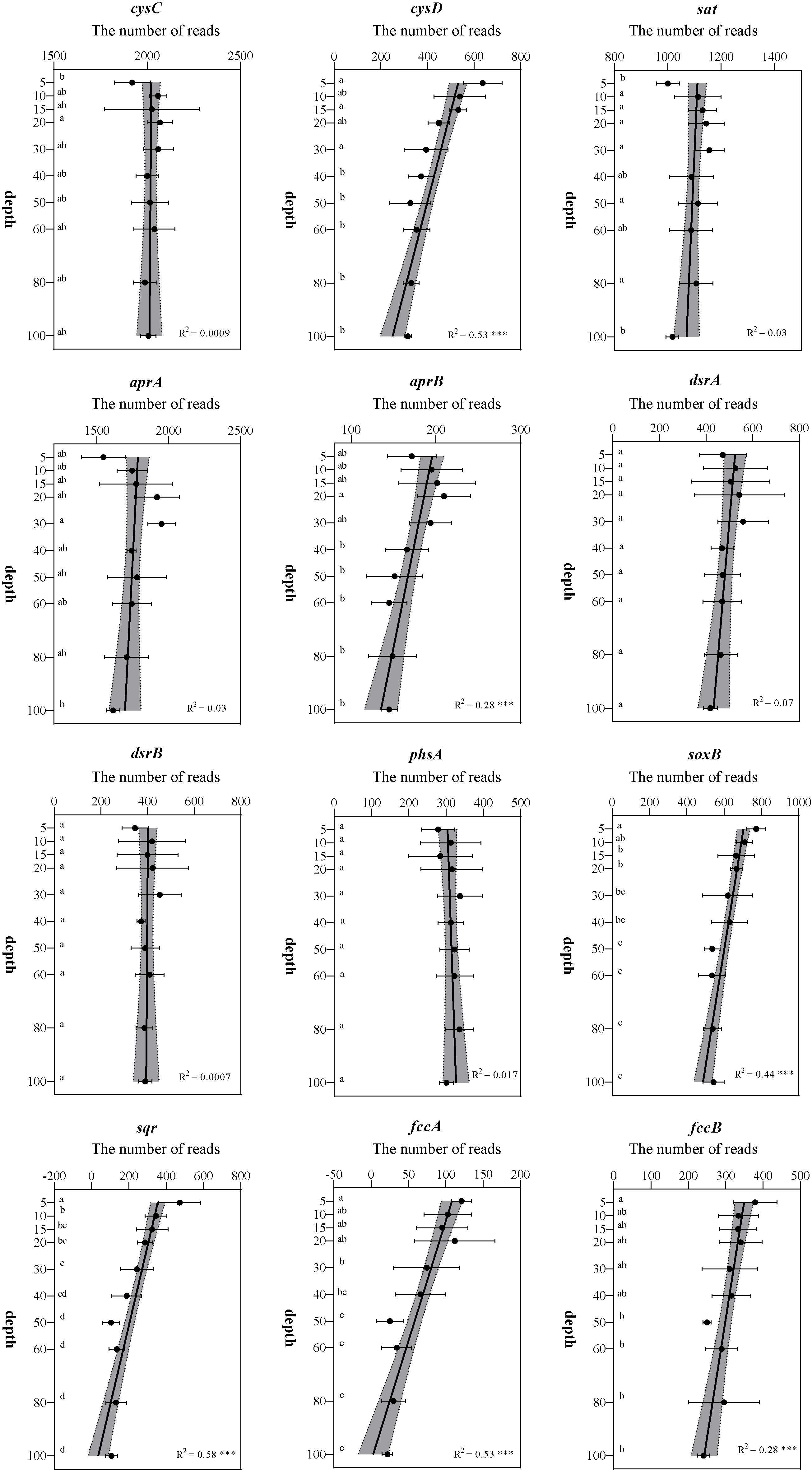

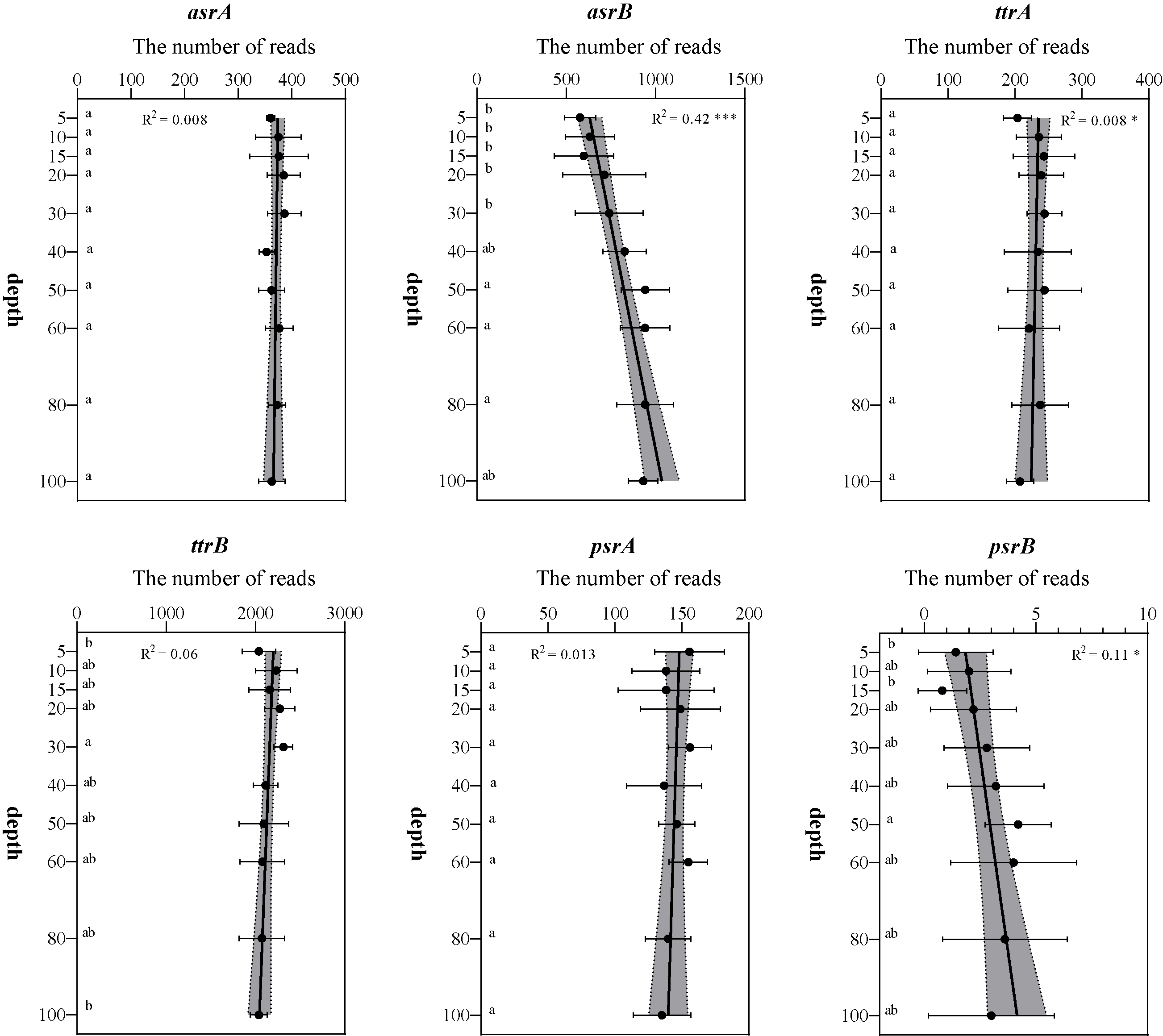


**Fig. S9** The vertical distribution of relative abundances of key gene families involved in sulphur cycling. Black lines and gray shaded areas represent linear regressions and 95% confidence intervals, respectively. R^2^ was obtained by linear regression analysis and significance levels are denoted with *(0.01 < *P* < 0.05), **(0.001 < *P* < 0.01) and ***(*P* < 0.001). Small letters mean a statistical significance (*P* < 0.05) among different depths.


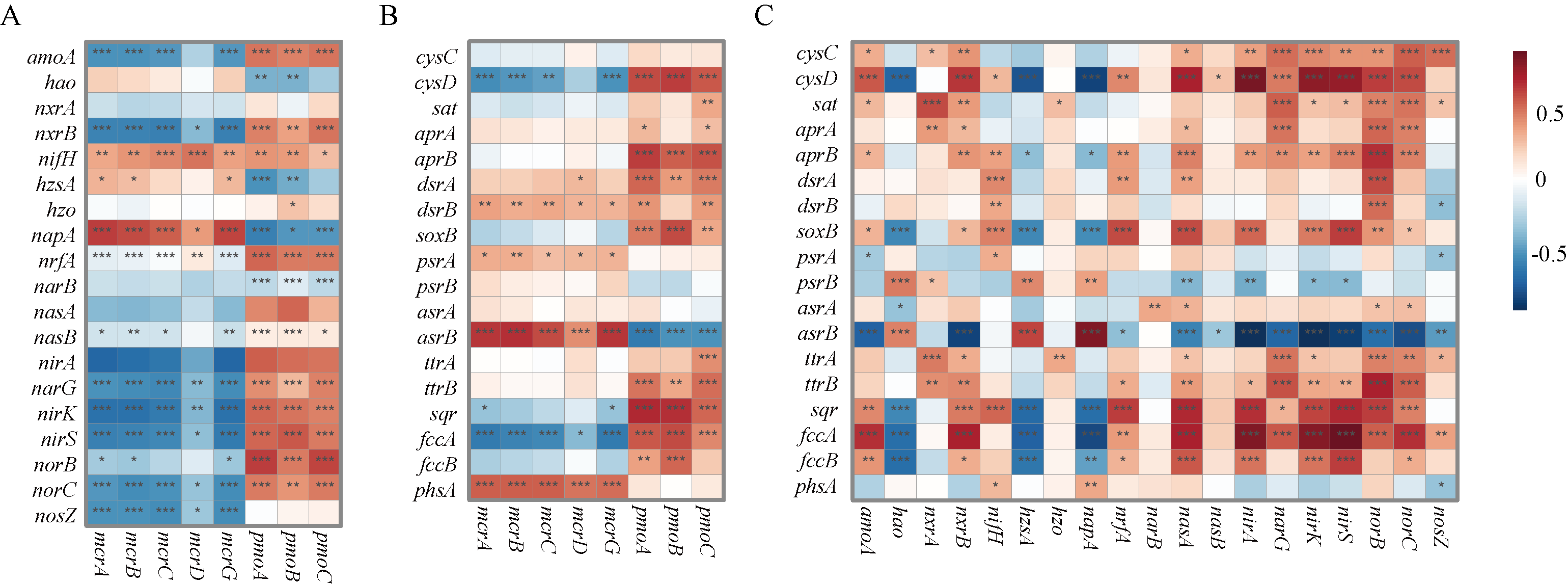


**Fig. S10** Correlations between the relative abundance of key gene families. Spearman analysis revealed the correlations between key gene families involved in N/S/CH_4_ cycling. a. N and CH_4_ cycling; b. S and CH_4_ cycling; and c. N and S cycling. ***: *P* < 0.001; **: 0.001 < *P* < 0.01; *: 0.01< *P* < 0.05.


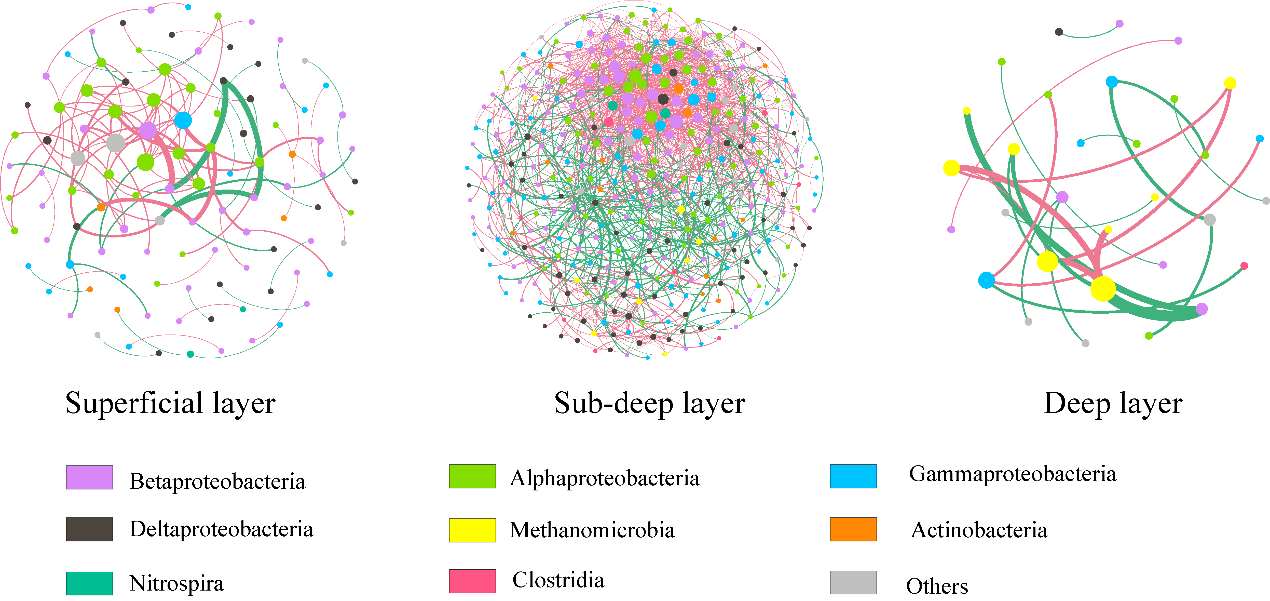


**Fig. S11** Methane, nitrogen and sulphur cycling co-occurrence networks of three sediment layers at the species level with OTUs colored by phylum/class. The size of each node is proportional to the number of connections (i.e., degree), and the thickness of each connection between two nodes (i.e., edge) is proportional to the value of correlation coefficients. Red edges indicate positive relationships between two individual nodes, while green edges indicate negative relationships.


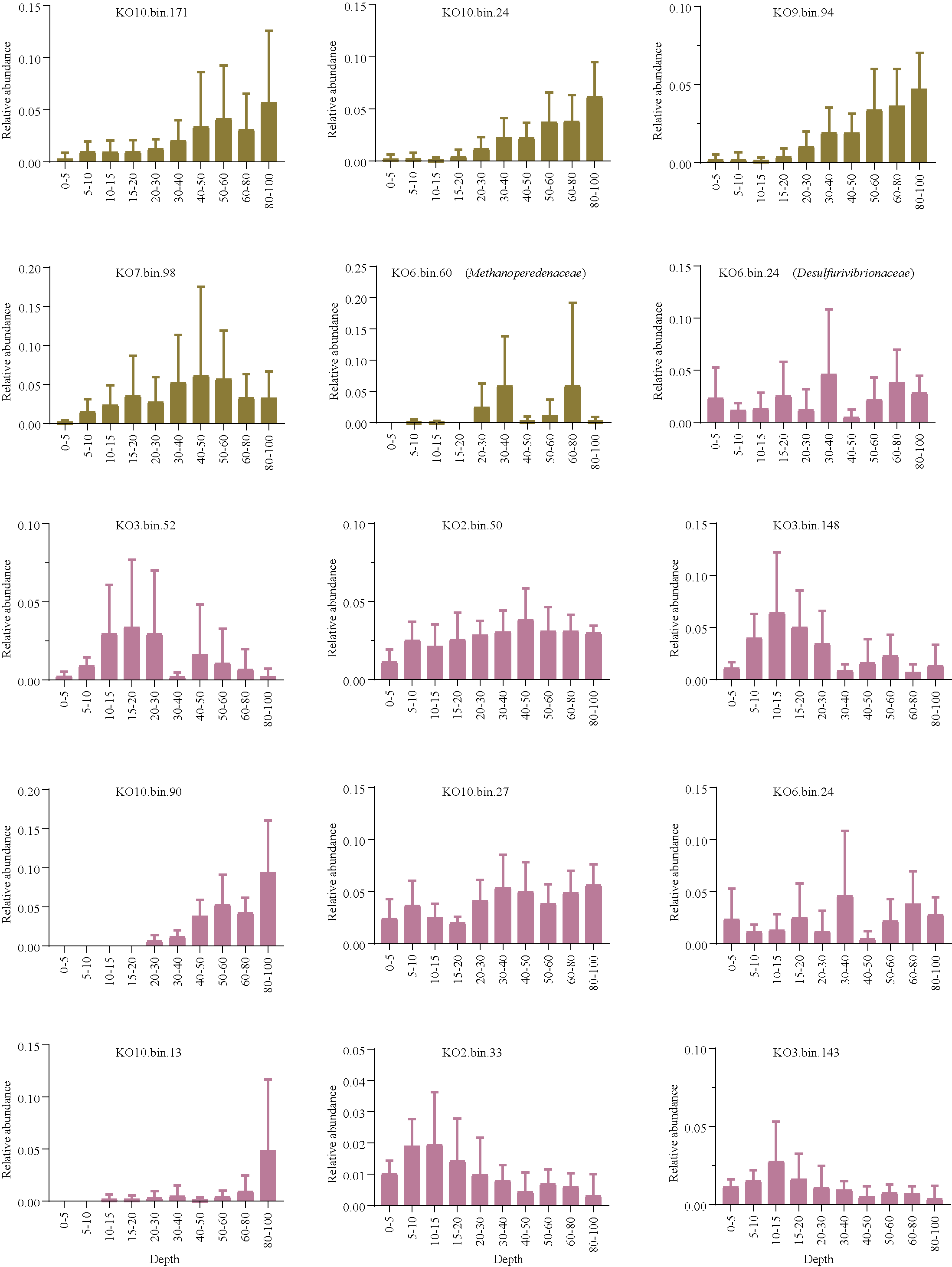


**Fig. S12** The vertical distribution of relative abundances of retrieved methanogen/ANME/SRB MAGs. The yellow columns refer to the relative abundance of methanogen/ANME and the purple columns refer to the relative abundance of sulphate-reducing bacteria.


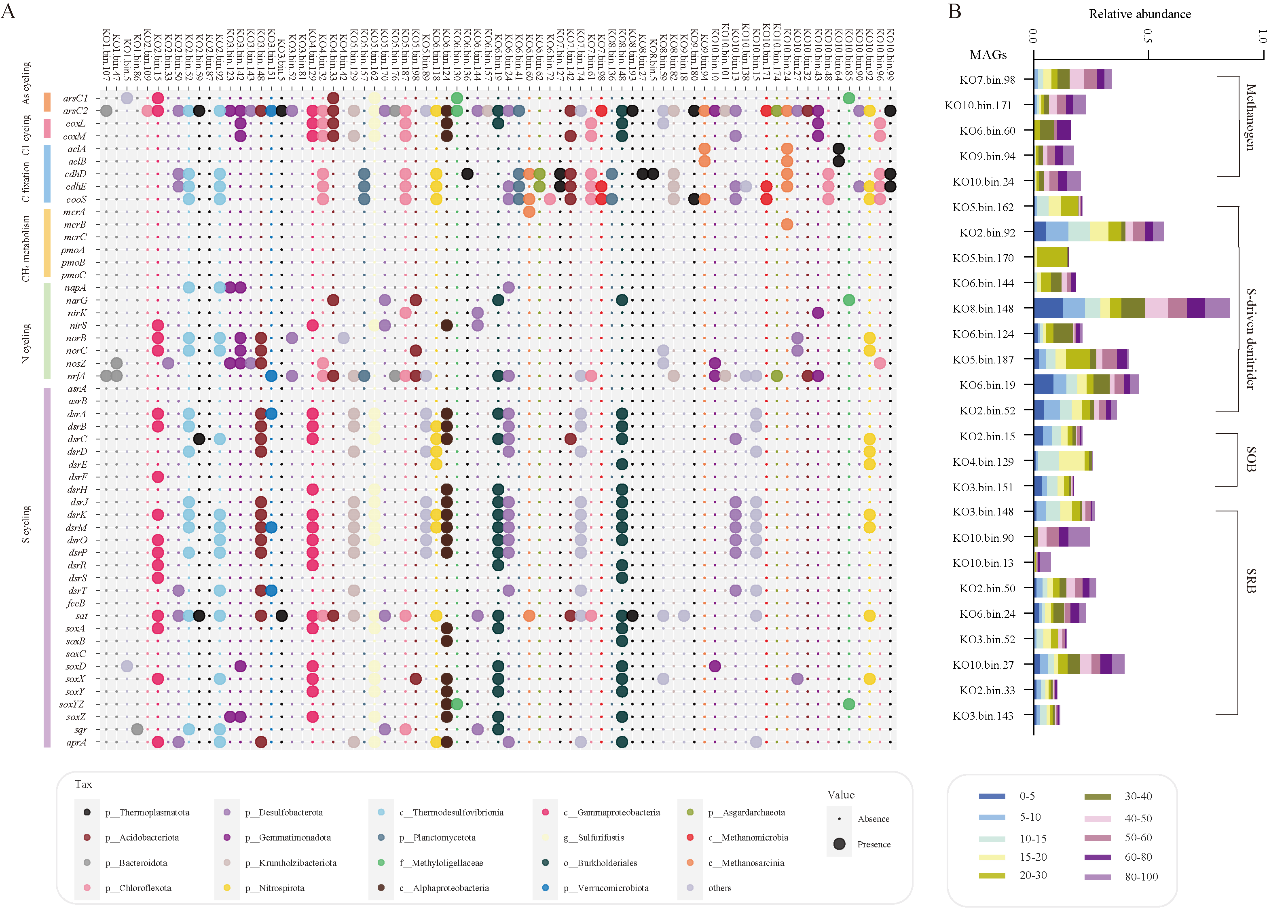


**Fig. S13** Metabolic profile of retrieved MAGs (A) and relative abundances of selected MAGs (B). Please refer to Additional file 2: Dataset S2 for more details.


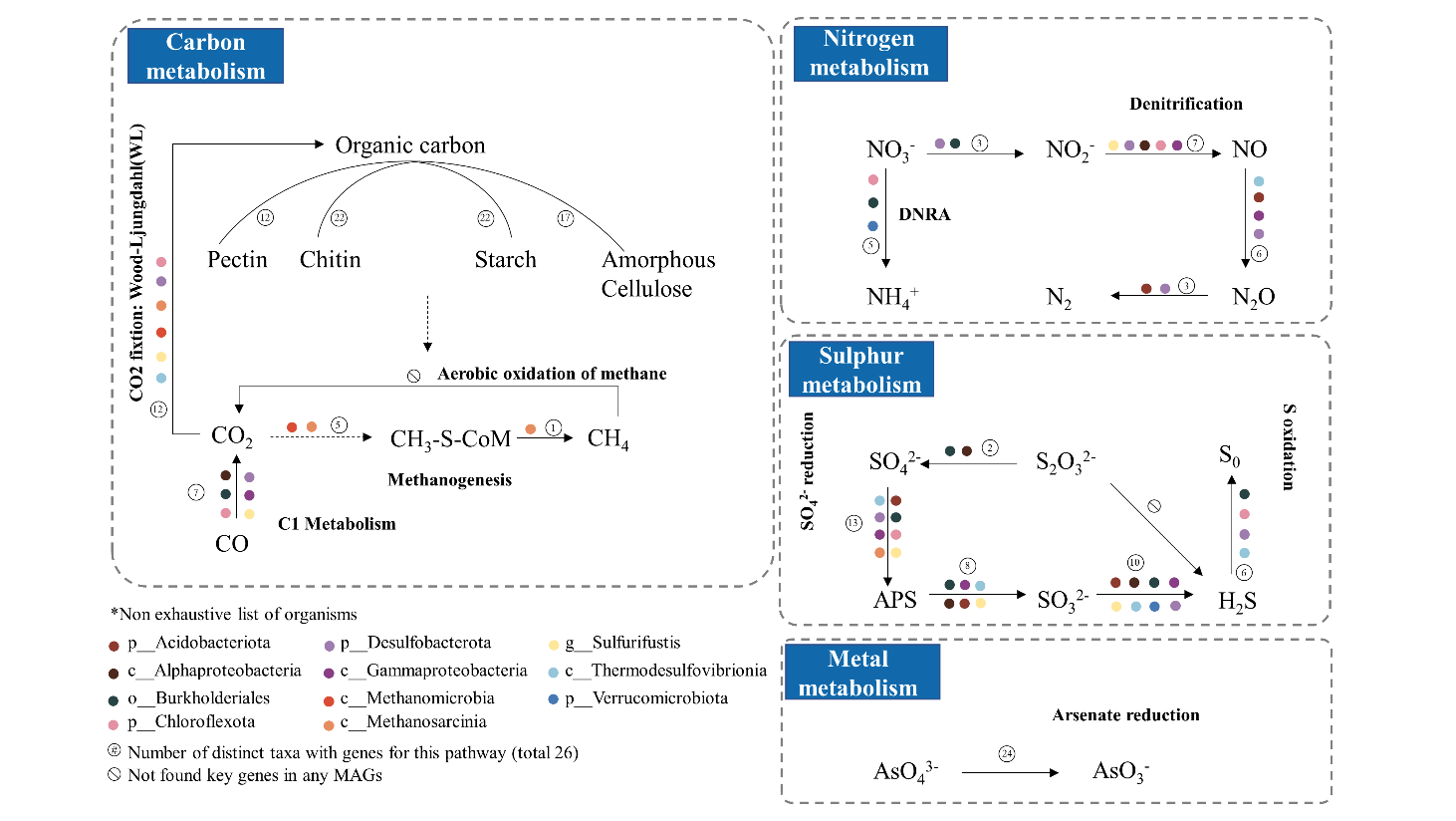


**Fig. S14** Carbon, nitrogen, sulphur and metal metabolisms involved in different selected lineages based on retrieved MAGs containing gene families involved in S oxidation, denitrification, dissimilatory nitrate reduction, sulphate reduction, methanogenesis and anaerobic oxidation of methane.
